# Learning-based segmentation of diffusion-weighted MR images with arbitrary *q*-space samplings

**DOI:** 10.1162/IMAG.a.1183

**Published:** 2026-06-02

**Authors:** Christian Ewert, David Kügler, Martin Reuter

**Affiliations:** German Center for Neurodegenerative Diseases (DZNE), Bonn, Germany; A. A. Martinos Center for Biomedical Imaging, Massachusetts General Hospital, Boston, MA, United States; Department of Radiology, Harvard Medical School, Boston, MA, United States

**Keywords:** deep learning, geometric deep learning, segmentation, diffusion MRI, *q*-space

## Abstract

Segmenting anatomical regions is a crucial step in many diffusion-weighted MRI (dMRI) workflows, such as region-of-interest analysis or anatomically-constrained tractography, which enable in vivo studies of brain microstructure and connectivity. However, convolutional neural networks (CNNs)—the foundation of most state-of-the-art segmentation models—require *structured inputs* with a *fixed* number of channels. This makes them ill-suited for dMRI, where acquisition protocols vary widely in q-space sampling—the number of measurements as well as their directions (b-vectors) and weightings (b-values)—resulting in *unstructured data* with inconsistent dimensionality across studies. As a consequence, the applicability of CNN-based methods is generally limited to the dataset on which they were trained. To address this, existing methods like DeepAnat and DDParcel rely on diffusion model fits, such as the diffusion tensor, to convert raw data into structured representations compatible with CNNs. While this enables broader applicability, it introduces lossy compression that can degrade performance. In this work, we propose a novel method that combines the geometric deep learning-based reconstruction framework DISCUS with the segmentation network VINN to directly map unstructured dMRI data to anatomical segmentations. Our segmentation approach is the first to achieve robust generalization across heterogeneous acquisition schemes using a single neural network without requiring diffusion model fits. Our approach generates the segmentation in minutes, whereas DeepAnat relies on the external FreeSurfer software, which runs for several hours. Additionally, we demonstrate generally superior segmentation performance of our approach across multiple datasets and acquisition settings with respect to DeepAnat, DDParcel, and SynthSeg.

## 1 Introduction

Diffusion MRI (dMRI) workflows extract valuable information on the brain’s macro- and microstructure but often rely on a segmentation of anatomical regions. More specifically, these segmentations enable region-of-interest analyses and inform tractography (R. E. Smith et al., 2012; Yendiki et al., 2011), that is, the reconstruction of the brain’s neural pathways from dMRI. Tractography, in turn, facilitates the in vivo study of brain architecture and connectivity (Yeh et al., 2020; F. Zhang et al., 2022).

One challenge for the segmentation of diffusion-weighted images (DWIs) is the gap between the heterogeneous nature of dMRI data featuring diverse q-space samplings and segmentation methods that are, by themselves, ill-equipped to accommodate this type of heterogeneous data. On one hand, diffusion MRI datasets differ tremendously in three important technical aspects: i) the number of DWIs (ranging from very few to several hundred), ii) the q-space acquisition scheme (single-, multi-shell, or grid-based), iii) as well as the details of the directions (b-vectors) and weightings (b-values) for which the DWIs are acquired. Due to this variation, dMRI data is highly *unstructured*. On the other hand, convolutional neural networks (CNNs)—state-of-the-art segmentation networks—require a *well-structured input* with a *fixed* number of channels and a consistent structure across multi-channel input samples. During training, a CNN’s parameters are tailored to the specific structure of the training dataset. Through these parameters, the CNN expects to find particular types of information in particular channels. In addition, the number of input channels is selected once and remains fixed. As a consequence of these two aspects, the trained CNN’s applicability to other datasets is severely limited, as they will practically always have a structure that differs from that of the training dataset (Ewert et al., 2024: Fig. 3f, Xiao et al., 2025: Fig. 2). That structure may differ in the number of images or include images for different directions (b-vectors) or weightings (b-values), conflicting with the requirement for a fixed channel number or a consistent channel structure, respectively.

To address this challenge, several works have used diffusion signal models, for example, the diffusion tensor (Basser et al., 1994), to map the unstructured raw diffusion data to a structured representation with a fixed number of consistent channels that a CNN segmentation network accepts as input (Bazin et al., 2011; H. Cheng et al., 2020; Efird et al., 2021; Ewert et al., 2021; Kebiri et al., 2023; Z. Li et al., 2023; Pinheiro et al., 2023; Theaud et al., 2022; Tregidgo et al., 2023; Wasserthal et al., 2018; F. Zhang et al., 2021, 2023). With these models, an unstructured diffusion MRI acquisition (the triplet of ordered b-vectors, b-values, and DWIs), featuring any number, order, directions, and weightings of measurements, can be transformed into a well-structured form. This well-structured form has a fixed channel number and a consistent channel structure and could, for example, comprise maps of fractional anisotropy (FA), mean diffusivity (MD), and the three eigenvalues derived from the diffusion tensor. However, while methods using these representations may have the potential for generalization as diffusion models can be fitted to data from a wide variety of acquisition schemes, the model fits are a lossy compression of the raw data, yielding lower segmentation quality (Ewert et al., 2021). Other approaches have used mean signal images, but this usually discards a lot of the directional contrast core to dMRI (H. Cheng et al., 2020; Efird et al., 2021; Ewert et al., 2021; Z. Li et al., 2023; Wang et al., 2020). Finally, some approaches use DWIs directly (Ewert et al., 2021; Golkov et al., 2016; Newlin et al., 2024), bypassing the lossy compression and enabling end-to-end learning, but these approaches do not generalize to other q-space acquisition schemes (Ewert et al., 2024; Xiao et al., 2025). Here, we focus on methods that may generalize across acquisition schemes.

We make the following contributions:

- We provide the first method that generates a dMRI segmentation with one network accommodating heterogeneous raw dMRI data acquisitions (selections of b-vectors, b-values, and signals)—without the need for model fits like the diffusion tensor. The DISCUS component provides a learned voxel-wise summary of the acquisition, avoiding the need for a lossy compression through model fits, and the VINN component generates the segmentation from this summary representation and a mean $b_{0}$ image.
- We demonstrate that this method generates high-quality segmentations generally outperforming the state-of-the-art methods *DeepAnat* and *DDParcel* as well as *SynthSeg* applied to mean $b_{0}$ images or FA maps. With the application to three different datasets, we show that our method generalizes across selections of single-shell measurements (ranging from 10 to 90 and differing in b-vectors) but also across other aspects (scanner manufacturers, spatial resolutions, and condition groups).

## 2 Related Work

### 2.1 dMRI segmentation

A wide variety of anatomical regions valuable for neuroimaging analyses have been segmented on dMRI or derived quantities: brain masks (Palanivelu et al., 2020), tissue types (H. Cheng et al., 2020; Golkov et al., 2016; Wang et al., 2020; F. Zhang et al., 2021), white matter tracts (Bazin et al., 2011; Kebiri et al., 2023; Wasserthal et al., 2018), lesions (Golkov et al., 2016; Liu et al., 2021; Saad et al., 2011; R. Zhang et al., 2018), individual structures, for example, the thalamus (Pinheiro et al., 2023), its nuclei (Tregidgo et al., 2023), and the hippocampus (Efird et al., 2021) as well as small (Theaud et al., 2022) or large sets of different anatomical regions (Ewert et al., 2021; Z. Li et al., 2023; F. Zhang et al., 2023). While most methods directly yield the desired segmentation, a recent shift has seen *DeepAnat* (Z. Li et al., 2023) synthesize T1w images from dMRI and successfully apply existing segmentation tools tailored to T1w images to obtain a segmentation, for example, FreeSurfer (Fischl, 2012). The backbone of choice for most of these methods is a U-Net (Ronneberger et al., 2015) or variants thereof.

### 2.2 Input representations

Related works propose three strategies to meet the requirement of neural networks, such as CNNs and MLPs, for well-structured inputs for heterogeneous diffusion data: i) diffusion model indices, ii) mean signal images, or iii) raw standardized DWIs as input to the segmentation network. Indices from diffusion model fits include those from the diffusion tensor (Basser et al., 1994) such as tensor components, fractional anisotropy, diffusivity, eigenvectors or -values (Bazin et al., 2011; Efird et al., 2021; Ewert et al., 2021; Z. Li et al., 2023; Pinheiro et al., 2023; Theaud et al., 2022; Tregidgo et al., 2023; F. Zhang et al., 2021, 2023), diffusion kurtosis imaging (Jensen & Helpern, 2010), that is, axial, radial, or mean kurtosis (F. Zhang et al., 2021), spherical harmonics (Kebiri et al., 2023), peaks of orientation distribution functions or their magnitudes (Theaud et al., 2022; Wasserthal et al., 2018), or other quantities (Z. Li et al., 2023). Several works have chosen mean DWIs (Efird et al., 2021; Z. Li et al., 2023) or mean images without diffusion-weighting, that is, images at b = 0 s/mm^2^ referred to as $b_{0}$ images (Ewert et al., 2021; Z. Li et al., 2023). Others have used DWIs directly (Ewert et al., 2021; Golkov et al., 2016; Newlin et al., 2024).

However, input representations obtained with a model fit may compress the information contained in the raw data, yielding lower segmentation performance as shown by Ewert et al. (2021) for the diffusion tensor. In addition, mean signal images do not use directional contrast (for $b_{0}$) or discard a large part of it (for $b_{>0}$ ), and the usage of DWIs directly implies a very limited generalization and application potential as the diffusion MRI datasets vary so widely (Ewert et al., 2024; Xiao et al., 2025).

### 2.3 Reconstruction network DISCUS

In the context of dMRI signal reconstruction (the task of reconstructing signals for additional directions from a set of measurements per imaging voxel), we have recently developed the *Geometric Deep Learning for Diffusion MRI Signal Reconstruction with Continuous Samplings* (DISCUS) method (Ewert et al., 2024). This method permits the flexible signal reconstruction for *arbitrary* b-vectors and b-values—termed *query vectors*—from an unstructured set of measurements (a triplet of b-vectors, b-values, and acquired signals)—termed *observation set*. DISCUS consists of an encoder and a decoder (see Fig. 1 in Ewert et al., 2024). The encoder creates a structured representation from unstructured inputs and converts information from a per-measurement basis in the observation set to a fixed-size latent vector (diffusion embedding). The decoder accepts an arbitrary query vector and the diffusion embedding vector as inputs and predicts the signal for the query vector, given the information in the embedding vector. The architecture of this method was designed particularly for dMRI data. It can accommodate any number of measurements and is invariant to the order of measurements in the observation set as well as to the sign of the b-vectors, taking into account the antipodal symmetry of the data. Furthermore, through data augmentation, DISCUS implements invariance to rotations of the basis of q-space (the joint rotation of vectors in the observation set and the query vector) and the method’s ability to reconstruct signals from a diversity of acquisitions, as it randomizes the number and choice of measurements in the observation set during training.

## 3 Materials and Methods

In this section, we describe the datasets, our method, its implementation details, the evaluation metrics, and the reference methods.

### 3.1 Data

#### 3.1.1 Datasets

We utilize diffusion MRI data ($b_{0}$ and $b_{1000}$ images) and T1w images from the Human Connectome Project (HCP) (J. Andersson et al., 2003; J. Andersson & Sotiropoulos, 2016; J. L. R. Andersson & Sotiropoulos, 2015; Essen et al., 2013; Feinberg et al., 2010; Fischl, 2012; Glasser et al., 2013; Jenkinson et al., 2002, 2012; Milchenko & Marcus, 2012; Moeller et al., 2010; Setsompop et al., 2012; Sotiropoulos et al., 2013; Xu et al., 2012), an in-house dataset acquired in an early version of the Rhineland Study protocol (Koch et al., 2024), and the Alzheimer’s Disease Neuroimaging Initiative (ADNI, adni.loni.usc.edu). Participants from all studies gave written informed consent in accordance with the ethical guidelines of the individual studies.

##### 3.1.1.1 HCP Young Adult

From the HCP Young Adult study, we create sex-balanced disjoint subsets of 250 participants for training, 50 for validation, and 100 for testing, primarily in the age range 22–35. The diffusion-weighted images were acquired at an isotropic spatial resolution of 1.25 mm for three shells at b-values of 1,000, 2,000, and 3,000 s/mm^2^ with 90 diffusion-encoding gradient directions per shell and 18 images with no diffusion-weighting ($b_{0}$) interleaved. The diffusion measurements were acquired for two opposite phase-encoding directions–improving the correction for susceptibility-induced distortions and providing higher-quality data. The T1w images were acquired at an isotropic spatial resolution of 0.7 mm. The data distributors performed the following pre-processing: the T1w images were bias field-corrected and rigidly aligned to the axes of MNI space. The dMRI data was corrected for EPI distortions with the *FSL* tool Topup (J. Andersson et al., 2003; S. M. Smith et al., 2004) as well as eddy currents and head motion with the *FSL* tool Eddy (J. Andersson & Sotiropoulos, 2016). In addition, they were registered to the T1w data with boundary-based registration.

##### 3.1.1.2 In-house dataset

From the superset of study participants with multi-shell acquisition, we create an external sex-balanced test set of 96 participants with a uniform distribution across three age ranges: 30–44 years (similar to the HCP), 45–59 years, and 60–85 years. All images were acquired on a 3 Tesla MRI scanner (MAGNETOM Prisma, Siemens Healthineers). The diffusion-weighted images were acquired at an isotropic spatial resolution of 1.5 mm for four shells at b-values of 700, 1,000, 2,000, and 3,000 s/mm^2^ with 6, 30, 36, and 48 diffusion-encoding gradient directions and a single phase encoding (Spin Echo EPI, TE: 93 ms, TR: 5,100 ms, echo spacing: 0.72 ms, and simultaneous multislice factor: 3). In addition, 7 images with no diffusion-weighting ($b_{0}$) were interleaved, and 4 $b_{0}$ images were acquired in the opposite phase encoding direction (posterior to anterior). The T1w images have an isotropic spatial resolution of 0.8 mm (MPRAGE with elliptical sampling (Brenner et al., 2014), TE: 2.94 ms, TR: 2,560 ms, TI: 1,100 ms, flip angle: $7^{\circ }$, echo spacing: 9.2 ms, CAIPIRINHA $1\times 3_{z1}$ ).

##### 3.1.1.3 ADNI

From the data of ADNI-4 (“Screening - New Pt” in the database), we assemble a dataset of 127 participants (67 male, 60 female) in the age range between 55 and 89 including different condition groups (Alzheimer’s disease (AD): 13, Mild cognitive impairment (MCI): 58, Healthy controls: 56 participants), and different scanners (3 Tesla Siemens Magnetom Prisma: 90, 3 Tesla General Electric (GE): 37 participants). The diffusion-weighted images were acquired at an isotropic spatial resolution of 2.0 mm (reconstructed to 0.9×0.9×2 mm on GE scanners) for three shells at b-values of 500, 1,000, and 2,000 s/mm^2^ with 6, 48, and 60 diffusion-encoding gradient directions (Spin Echo EPI, TE: 82 ms, TR: 3,400 ms, and simultaneous multislice factor 3). In addition, 13 images with no diffusion-weighting ($b_{0}$) were interleaved. For the Siemens scans, 7 images in the opposite phase-encoding direction were also acquired (anterior to posterior, 1 $b_{0}$ image, 6 $b_{2000}$ images) while susceptibility corrections were applied directly on the scanner for images from the GE scanners (Arani et al., 2024). The T1w images have an isotropic spatial resolution of 1.0 mm (MPRAGE, TE: min full, TR: 2,300 ms, TI: 900 ms, flip angle: $9^{\circ }$). Data used in the preparation of this article were obtained from the Alzheimer’s Disease Neuroimaging Initiative (ADNI) database (adni.loni.usc.edu). The ADNI was launched in 2003 as a public-private partnership, led by Principal Investigator Michael W. Weiner, MD. The original goal of ADNI was to test whether serial magnetic resonance imaging (MRI), positron emission tomography (PET), other biological markers, and clinical and neuropsychological assessment can be combined to measure the progression of mild cognitive impairment (MCI) and early Alzheimer’s disease (AD). The current goals include validating biomarkers for clinical trials, improving the generalizability of ADNI data by increasing diversity in the participant cohort, and to provide data concerning the diagnosis and progression of Alzheimer’s disease to the scientific community. For up-to-date information, see adni.loni.usc.edu.

#### 3.1.2 Preprocessing

We implement a preprocessing pipeline that includes the steps outlined in the DeepAnat paper (Z. Li et al., 2023) and employ it to preprocess the images from all participants and datasets. This pipeline consists of four steps: bias field correction, registration and resampling, mask generation, and segmentation with FreeSurfer and FastSurfer. The inputs to this pipeline are susceptibility- and head motion-corrected diffusion images at $b_{0}$ and $b_{1000}$ , the corresponding b-vectors and b-values, and a T1w image. The outputs are biasfield-corrected diffusion images with corresponding b-vectors and b-values, a biasfield-corrected T1w image in alignment with the diffusion images, as well as segmentation maps and brain masks. All images have the same alignment and spatial resolution of 1 mm.

##### 3.1.2.1 DICOM conversion and Gibbs ringing correction

ADNI data is converted from the DICOM to the NIfTI format with the *dcm2niix* tool (Gorgolewski et al., 2011; X. Li et al., 2016), and dMRI data is corrected for Gibbs ringing (Kellner et al., 2015; Tournier et al., 2019).

##### 3.1.2.2 dMRI corrections for susceptibility distortion, eddy current distortion, and head motion

The DeepAnat preprocessing pipeline requires diffusion data corrected for susceptibility and eddy current distortions as well as head motion. For the HCP dataset, the data distributors have already applied these corrections. For the in-house and ADNI datasets, we modify an in-house processing pipeline (Tobisch et al., 2018) –similar to that of the HCP–also building on the FSL tools Topup (J. Andersson et al., 2003; S. M. Smith et al., 2004) and Eddy (J. Andersson & Sotiropoulos, 2016), where $b_{0}$ images of both phase encoding directions are supplied to Topup, and Eddy corrects motion across slices and volumes. For the ADNI data, Topup is only applied to the Siemens scans, as GE scans have already been susceptibility-corrected in the scanner (Arani et al., 2024).

##### 3.1.2.3 Bias field correction

For the dMRI data of all datasets, we obtain a mean $b_{0}$ image from three $b_{0}$ images, calculate its bias field with the *Statistical Parametric Mapping (SPM)* suite (Version 12, Wellcome Centre for Human Neuroimaging, London, UK; https://www.fil.ion.ucl.ac.uk/spm/), and remove the bias field from the mean $b_{0}$ image and the DWIs. While the data distributors have already applied the bias field correction for the T1w image of the HCP dataset, we utilize SPM for the correction of the T1w image of the in-house and ADNI datasets.

##### 3.1.2.4 Registration and resampling

In this step, T1w images are registered to the mean $b_{0}$ image. To obtain the best possible registration, we obtain a bias field-corrected *high-quality* mean $b_{0}$ image from all available $b_{0}$ images (18/7/13 for the HCP/in-house/ADNI datasets), which we only use as the registration target. We register the T1w images to the target high-quality mean $b_{0}$ images with an affine registration obtained with *NiftyReg*’s *reg_aladin* tool (Modat et al., 2014). For the HCP dataset, this registration refines the pre-registration by the data distributors as documented in the DeepAnat paper. For the other datasets, this registration exclusively aligns the images of the two contrasts. To obtain images at the desired spatial resolution of 1 mm, we obtain a 1 mm target grid with *FastSurfer*’s *conform.py* and resample the diffusion data to that grid using *NiftyReg*’s *reg_resample*. We align the T1w image to the resampled diffusion images, combining the obtained registration and the resolution mapping in a single resampling step.

##### 3.1.2.5 Masks

We obtain a brain mask for the diffusion data with the tissue probability maps obtained with the SPM tool in the bias field correction step, which we also resample to 1 mm with *NiftyReg*’s *reg_resample*. More specifically, we threshold the sum of white matter, gray matter, and CSF probabilities at 0.95, perform binary hole-filling, dilate twice, and perform hole-filling again. We follow the same strategy to obtain T1w brain masks for the in-house and ADNI datasets, where SPM supplies the required tissue probabilities. In contrast, for the T1w images of the HCP dataset, we do not have access to the tissue segmentations, as the bias field correction was applied by the data distributors. For these images, we obtain a mask using the *Brain Extraction Tool (BET)* of the *FSL* suite (S. M. Smith, 2002; S. M. Smith et al., 2004) with a fractional intensity parameter of 0.3, allowing for larger brain outlines compared to the default. Importantly, for all methods, we only use diffusion-based masks as opposed to T1w masks at inference time, ensuring that no hidden T1w dependency is introduced.

##### 3.1.2.6 Reference segmentations

We obtain anatomical segmentations of T1w images with *FreeSurfer* 7.3.2 (Fischl, 2012) and, for the HCP dataset, additionally, with *FastSurfer* (Henschel et al., 2020), including subcortical labels and the cortical parcellation according to the “Desikan–Killiany–Tourville” (DKT) atlas (Desikan et al., 2006; Klein & Tourville, 2012). We follow the label mapping as done in FastSurfer (Henschel et al., 2020); for example, we merge small vessels with the adjacent white matter and, additionally, merge the corpus callosum sub-labels into one combined label.

### 3.2 Method

#### 3.2.1 Architecture

**Fig. 1.**
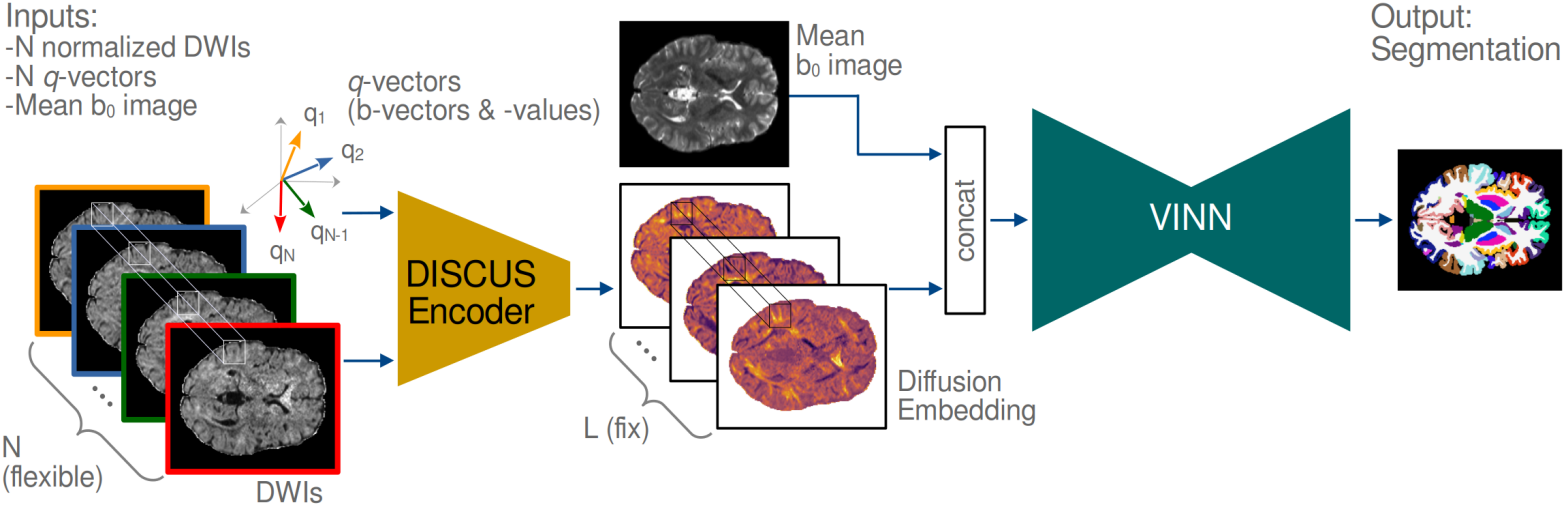
Method Overview: Our DISCUS+VINN method establishes an anatomical segmentation (right) for an arbitrary choice and flexible number N of diffusion-weighted images (left) and consists of two components: the DISCUS encoder (orange) and the segmentation network VINN (turquoise). The DISCUS encoder transforms the *unstructured* raw diffusion acquisitions (N-triplet of b-vectors, b-values, and DWIs) into a *well-structured* diffusion embedding representation with a *fixed* number L of channels and a consistent channel structure. This diffusion embedding is concatenated with a mean $b_{0}$ image and serves as input to the VINN segmentation network.

Our DISCUS+VINN method generates anatomical segmentations from unstructured diffusion data with a flexible number of images, that is, DWIs for an arbitrary choice and number of b-vectors and b-values. The architecture consists of two components: the encoder of the signal reconstruction network DISCUS (Ewert et al., 2024) and the segmentation network VINN (Henschel et al., 2022)—an extension to the U-Net architecture (see Fig. 1). The purpose of the DISCUS encoder is to transform the *unstructured diffusion data* with a *flexible* number of images—incompatible with any U-Net—into a compatible one, that is, a *well-structured representation* with a *fixed* number of channels and a consistent channel structure. In contrast, the purpose of VINN is to generate the segmentation based on the diffusion embedding from the DISCUS encoder concatenated with a mean $b_{0}$ image and provide improved scale generalization. DISCUS was originally designed for flexible angular super-resolution, where the diffusion embedding vector represents the information of the measurements per voxel and serves as the basis for the prediction of additional signals. Because this concept has been successfully applied across acquisition schemes and samplings, we hypothesize that these embeddings also serve as a comprehensive representation for image segmentation.

##### Details

To segment a 3D volume of shape (W,H,D,C) with width W, height H, depth D, and channels C into a segmentation volume with S classes, the VINN method follows a 2.5D approach for 3D segmentation. The training phase comprises the independent training of three fully 2D-convolutional networks on axial, coronal, and sagittal *slices*. At inference, per-view predictions are assembled into 3D volumes and combined across views. As for any 2D U-Net (also VINN), the input must be a tensor of shape (W,H,C) with width W, height H, and a predefined fixed number of channels C. We can satisfy the requirement for a predefined dimension C with DISCUS: we transform the slice of shape (W,H,N) with a varying number of diffusion measurements N into an embedding of shape (W,H,L) with a fixed embedding length L. This embedding is concatenated with the corresponding slice of the mean $b_{0}$ image and its two immediate neighbors—satisfying the requirement by fixing C=L+3 . We create the fixed-size embedding with DISCUS as follows: In contrast to VINN, DISCUS natively works on a *voxel* basis—not on 2D or 3D images. The DISCUS encoder expects an ordered triplet of b-vectors, b-values, and normalized signals for each voxel, each with flexible, yet same length N, and computes a diffusion embedding of fixed length L. To reduce the computational effort for a given slice (W,H), we mask the voxels by the brain mask and only execute DISCUS for voxels inside the brain mask. We then remap voxels into the slice according to the brain mask and fill “empty” voxels with zero. Due to memory constraints, we do not add neighboring slices for the diffusion embedding block as we do for the mean $b_{0}$ image.

#### 3.2.2 Training

In this subsection, we describe the training details of our method. We train DISCUS+VINN in two phases. In the first phase, DISCUS is pre-trained with the reconstruction objective. In the second phase, the pre-trained DISCUS encoder is combined with VINN, which is trained with the segmentation objective. After the outline of the two training phases, we provide details about data augmentation during training and list alternative training schemes.

##### 3.2.2.1 Phase 1: DISCUS pre-training

We pre-train DISCUS in the reconstruction context as outlined in the original paper (Ewert et al., 2024). This means taking a dMRI dataset featuring M diffusion measurements (M=90 DWIs for the HCP) and letting the DISCUS network reconstruct those M signals from a variety of smaller subsets with N<M signals. DISCUS reconstructs these signals *per imaging voxel* as opposed to a 2D or 3D image. For DISCUS to learn the reconstruction from various inputs, we randomly sample N of M measurements during training. We use the reconstruction objective to adapt network parameters in the DISCUS encoder and decoder, that is, the minimization of mean squared loss between predictions and reference signals.

###### Details

While the pre-training follows the original paper in most aspects (Ewert et al., 2024), we make the following changes. We pre-train on signals from white matter, gray matter, and CSF voxels (based on SPM) of 20 participants (10 male, 10 female) included in the HCP training set and validate on data from 2 participants (1 male, 1 female) included in the HCP validation set. Furthermore, DISCUS is pre-trained for 100 epochs, where the initial learning rate of 0.005 was decreased after 50 epochs to 0.0005.

##### 3.2.2.2 Phase 2: VINN training

We train the VINN with the segmentation objective, combining a median frequency-weighted logistic loss with edge-focus and a Dice loss, using the FreeSurfer-based segmentations described in Section 3.1.2.6 as target labels. As the inputs to the VINN are the DISCUS diffusion embeddings, DISCUS is part of this training; however, DISCUS model weights are frozen during this training phase, and this objective only affects parameter updates in VINN.

###### Details

We follow the original paper (Henschel et al., 2022) and use the default VINN implementation parameters^1^ with few changes: We keep the learning rate constant at 0.0001 for convergence after 70 of 80 training epochs, double feature channels for increased capacity, and use a batch size of 2 per GPU while employing gradient checkpointing in VINN.

##### 3.2.2.3 Augmentations

We implement desirable network properties with data augmentations. More specifically, we aim for network invariance to q-space samplings and q-space rotations for the DISCUS component and image scale equivariance for VINN (Ewert et al., 2024; Henschel et al., 2022).

###### q-space samplings

As motivated in the introduction, we desire the segmentation to be (as) independent (as possible) of the number, choice, and order of measurements (b-vector, b-value, DWI triplets) used as input. We implement this by randomizing the number and choice of measurements supplied as input for DISCUS both during the *DISCUS pre-training* and the *VINN training*. As DISCUS is by design invariant to the input order, specialized augmentation is redundant. During *DISCUS pre-training*, we apply q-space sampling augmentation per voxel following the per-voxel training scheme. In contrast, during *VINN training*, training is performed per image slice, and the sampling augmentation is applied identically for each voxel of that slice. In both cases, we supply between five and 90 (all) measurements with a uniform distribution between those bounds.

###### q-space rotations

We desire the resulting segmentation to be invariant to rotations of q-space. During *DISCUS pre-training*, where each voxel is considered an independent training sample, we apply uniform random rotations jointly to the observation and query vectors (see also Section 2.3).

###### Scale augmentation

During *VINN training*, we randomize the scale factors in the interpolation layers following a normal distribution to make the network equivariant to small-scale changes (see Henschel et al., 2022).

##### 3.2.2.4 Alternative training schemes

In a method ablation (Section 4.1.1), we test multiple alternatives to train DISCUS+VINN. In addition to separate *DISCUS pre-training* and *VINN training* phases, we consider the following two strategies:

###### Training for segmentation from scratch

We train DISCUS+VINN jointly from scratch with the segmentation objective (no pre-training with the reconstruction objective).

###### Multi-task training for segmentation and reconstruction

DISCUS+VINN, augmented by the DISCUS decoder, is trained from scratch with both the segmentation and the reconstruction objectives. For reconstruction, the DISCUS decoder predicts signals from the diffusion embeddings of the encoder, and the reconstruction objective affects the parameters of both the DISCUS encoder and decoder. In contrast, VINN generates the segmentation based on the diffusion embedding, and the segmentation objective yields parameter updates of VINN (like in all other training schemes), and here, additionally, of the DISCUS encoder. In practice, we execute two forward passes with parameter updates applied jointly: A reconstruction forward pass with voxel-based rotation and sub-sampling augmentations, and a segmentation forward pass without rotation, but with slice-based sub-sampling augmentation.

###### Details

Due to computational constraints, we train these versions only for the coronal view (not all three views), with reduced feature dimensions in DISCUS (main feature dimension 64) and VINN (feature dimension from reference implementation), and for 70 (instead of 80) epochs. For fair comparison, we also train the default method in this ablation setting. For all three versions, we apply gradient checkpointing in both network components.

### 3.3 Reference methods

#### 3.3.1 DeepAnat

We compare with *DeepAnat* (Z. Li et al., 2023), a state-of-the-art method for dMRI-based segmentations, which we retrain on the HCP training set. We follow the U-Net approach in their paper to synthesize T1w images and obtain segmentations based on those images with *FreeSurfer* 7.3.2 (Fischl, 2012). In its default setting, the DeepAnat workflow uses the diffusion tensor model to convert a mean $b_{0}$ image obtained from three $b_{0}$ images and the first 30 (of 90) DWIs at $b_{1000}$ for the HCP dataset to the network inputs. These network inputs consist of mean images for $b_{0}$ and $b_{1000}$ , eigenvalue maps from the diffusion tensor, and “standardized images” obtained from sampling the tensor model along a priori-defined directions. Although the paper does not investigate DeepAnat’s flexibility across q-space samplings, we note that the method should generalize, as the diffusion tensor approach can produce a well-structured input with a fixed number of channels and a consistent channel structure from a variety of q-space samplings.

##### Details

DeepAnat requires a brain mask for training and inference. We use separate masks for diffusion and T1w data to account for a local lack of correspondence between images, e.g., caused by different field-of-views or signal reduction and blurring in diffusion MRI. For the details on how these masks were obtained, see Section 3.1.2.5. During inference, we only apply the diffusion mask as a T1w mask cannot be considered available, and obtain the mean and standard deviation scaling factors on a randomly selected training participant, as suggested by the DeepAnat implementation. For all other parameters of the DeepAnat training, we use the default values as defined in their published implementation (including the mirroring augmentation)^2^.

##### Segmentation with FreeSurfer

With the default FreeSurfer pipeline, we encountered many failure cases when trying to obtain segmentations from the synthetic T1w images obtained with DeepAnat for HCP data and the default q-space sampling (26 of 50 cases of the validation set). Specifically, failure cases include both the absence of output and failed segmentations without any overlap of the brain outlines of the prediction and reference. To reduce the number of failure cases, we modify the FreeSurfer pipeline as follows: 1. Bypass the bias field correction for the Talairach registration and further processing, as well as the skull-stripping step. Both can be assumed obsolete for the synthesized T1w images, which do not contain the skull, and the generating network was trained and evaluated on bias field-corrected T1w images. 2. Run FreeSurfer a second and third time for failure cases. These two steps reduce the number of failure cases on the validation set and the default q-space sampling for DeepAnat to zero. We still observe one failure case (female, from a total of 100 cases) in the HCP test set for the sparse sampling of 10 DWIs, one failure case (male, 76 years old, from a total of 96 cases) in the in-house dataset, and one failure case (female, 86 years old, MCI, GE, from a total of 127 cases) in the ADNI dataset. For a fair comparison, we apply the same mappings described in Section 3.1.2.6 to the FreeSurfer segmentations based on the synthesized T1-weighted images.

#### 3.3.2 DDParcel

We compare with DDParcel (F. Zhang et al., 2023), another state-of-the-art method for diffusion MRI segmentation. DDParcel generates the segmentation based on an input stack consisting of diffusion tensor-derived quantities: fractional anisotropy, mean diffusivity, and the second and third largest eigenvalues. We use the published parameter checkpoint^3^ of their method, also trained on data of the Human Connectome Project, leaving all settings—except for two—at their default values. First, we found that the brain masking in the DDParcel workflow does not reliably segment brain tissue, often including vast amounts of skull tissue and degrading performance. Therefore, we supply the mask that we generated during preprocessing (see Section 3.1.2.5). Second, the registration to the template was found to be unreliable, cutting off vast amounts of brain tissue in some cases. We improve this by increasing the sampling percentage from its default of 0.2% to 20%. For a fair comparison, we apply the same mappings described in Section 3.1.2.6 to the DDParcel segmentations.

#### 3.3.3 SynthSeg

In addition, we compare our method with SynthSeg—a method capable of segmenting MRI images of any contrast and/or resolution (Billot et al., 2023). Specifically, we generate two segmentations with SynthSeg applied to different inputs: 1) the mean $b_{0}$ image and 2) the fractional anisotropy (FA) map derived from the diffusion tensor (fitted with FSL’s *dtifit* S. M. Smith et al., 2004). We use the pretrained implementation implemented in FreeSurfer via *mri_synthseg* (Version 2.0). In contrast to other reference methods, we do not apply a brain mask, that is, we supply the mean $b_{0}$ image with the skull outline and fit the diffusion tensor on the entire image. For a fair comparison, we apply the same mappings described in Section 3.1.2.6 to the SynthSeg segmentations.

### 3.4 Evaluation

#### 3.4.1 Performance metrics

We evaluate predicted segmentation maps with respect to the reference segmentation maps with two metrics: Dice Similarity Coefficient (DSC) and 99th percentile of the Hausdorff distance (HD99) (for definitions see Figures *SN 3.5.* and *SN 3.27.* in Maier-Hein et al. (2024)). For central evaluations, we conduct one-sided Wilcoxon signed-rank tests (Wilcoxon, 1945) on pairwise metric differences across regions and participants between ours and any competing approach, under the null hypothesis that our method does not outperform the competing approach.

#### 3.4.2 q-Space samplings

To test the methods’ performance concerning different samplings of q-space for the HCP dataset, we obtain sub-samplings with 10, 30, and 90 b-vectors (from the available 90 b-vectors at $b_{1000}$ ), representing sparse, medium-quality, and high-quality diffusion MRI acquisitions. We obtain these samplings via *DMRI Tool* (J. Cheng et al., 2018), aiming to maintain maximum angular coverage. In addition, we also evaluate the methods on the q-space sampling used for DeepAnat training, which consists of the first 30 of 90 measurements. This set differs from the 30 vectors above and is denoted by 30*. For the in-house and ADNI datasets, we apply no sub-sampling as the datasets contain only 30 and 48 vectors, respectively. These samplings provide a single selection (in-house and ADNI datasets) or multiple selections of DWIs (HCP dataset) per participant.

#### 3.4.3 Labels

For the evaluation, we *merge* and *group* selected label sets to ensure consistent label definitions across methods and to present the results in a compact form. Merging is performed before metric computation, while grouping aggregates calculated metrics across regions. By merging, we obtain the following labels for prediction and reference segmentation maps: i) one cortical region per hemisphere by merging the contained regions, ii) a *choroid plexus with ventricles* region merging choroid plexus and lateral ventricle labels across hemispheres, and iii) a combined *white matter* label merging the white matter labels of both hemispheres with white matter hypointensities and the corpus callosum label(s) (if present). We build the following region labels with grouping: white matter (merged region above), subcortical gray matter, cortical gray matter (merged labels per hemisphere), ventricles and cerebrospinal fluid (CSF) (termed “Vent-CSF”), incl. merged *choroid plexus with ventricles* region above, and cerebellar regions. More specifically, and for each group, we average the metrics across contained regions and report the performance distribution over participants. All differences in label definitions across methods can be resolved with merging, except for one. The SynthSeg method differs substantially in its CSF definition from FreeSurfer’s definition, precluding a direct comparison. Therefore, we do not report the performance of the Vent-CSF regions for SynthSeg.

## 4 Experiments & Discussion

In this section, we present and discuss the results of our method ablation and compare our DISCUS+VINN with state-of-the-art reference methods on the HCP, in-house, and ADNI datasets. In all evaluations, we measure the performance of the methods with the Dice Similarity Coefficient (DSC) and the 99th percentile of the Hausdorff distance (HD99) for five different region groups: white matter, subcortical gray matter (GM), cortical gray matter, ventricles and cerebrospinal fluid (termed Vent-CSF), as well as cerebellar regions. For the evaluations in this section, we use FreeSurfer segmentation labels as reference unless otherwise stated.

### 4.1 Ablations

#### 4.1.1 Training strategies

**Fig. 2.**
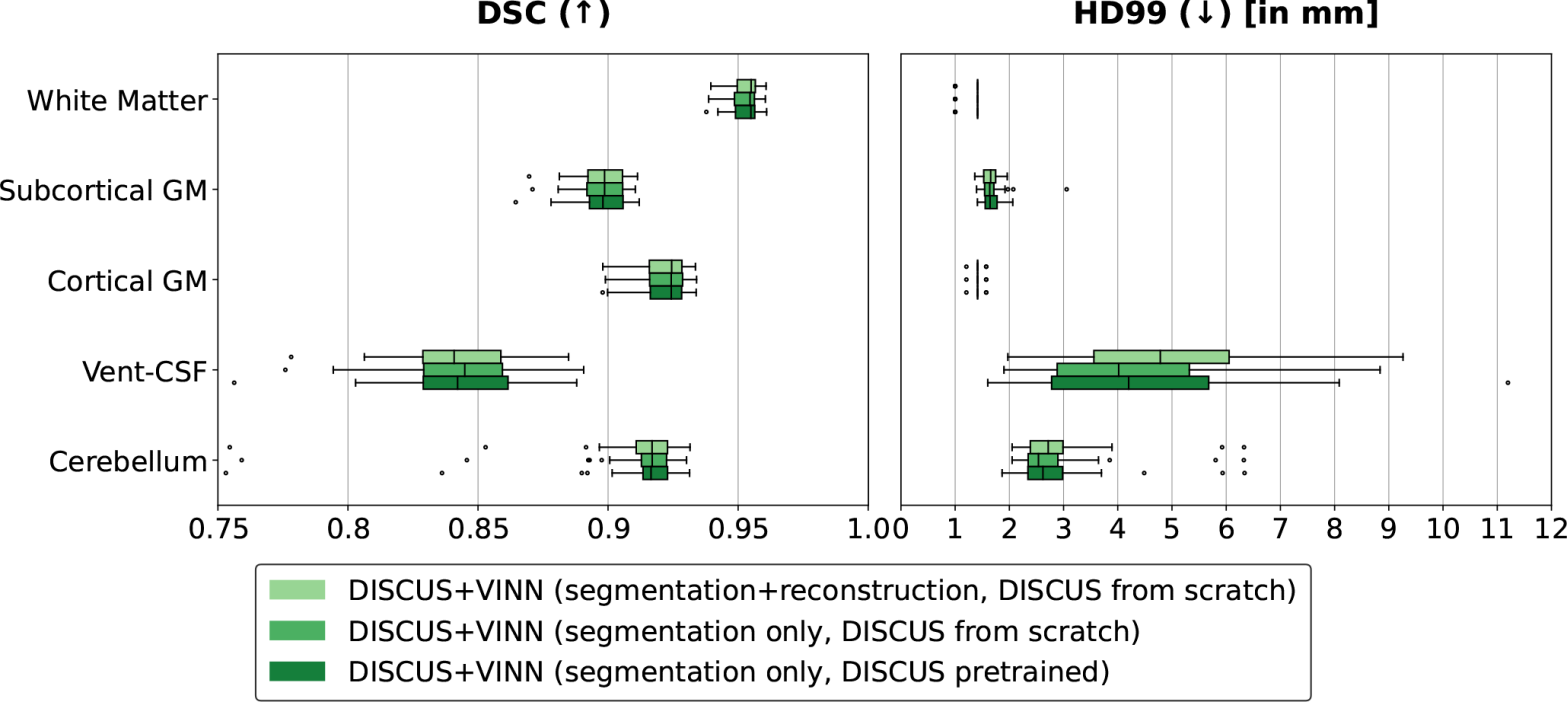
Ablation of training strategies: Comparison of the segmentation performance for different training versions of our DISCUS+VINN method evaluated on the HCP validation set. We compare the performance for two metrics: the Dice Similarity Coefficient (DSC) and the 99th percentile of the Hausdorff distance (HD99), across five region groups: White matter, subcortical and cortical gray matter (GM), ventricles and cerebrospinal fluid (Vent-CSF), and cerebellar regions, and with respect to FreeSurfer reference segmentations.

In order to determine the impact of the DISCUS+VINN training strategy on segmentation performance, we train three different versions in limited settings (see Section 3.2.2.4) on the HCP training set and evaluate them on the HCP validation set (see Fig. 2): 1. a joint training of DISCUS+VINN with both a segmentation and a reconstruction objective, 2. a joint training of DISCUS+VINN with the segmentation objective only, and 3. a pre-training of DISCUS with the reconstruction objective and a training of VINN with the segmentation objective (frozen weights for DISCUS). For the three method versions, we observe a high segmentation performance across the board, but in comparison, slightly lower performance for sometimes small and isolated—and therefore challenging—Vent-CSF regions. We observe highly similar performances of the three versions for both metrics and all five region groups.

We expected the joint training to be more effective. However, the good performance of the pre-trained model can be explained by the extended use of data augmentation in voxel-wise pre-training (see Section 3.2.2.3). Another factor relevant to method selection is the GPU memory demand: the joint training for segmentation and reconstruction requires the most GPU memory, with the joint training for segmentation requiring the second most. With both versions, feature maps for DISCUS *and* VINN have to be kept in memory for the backpropagation. In contrast, the version with DISCUS pre-training requires substantially less GPU memory during segmentation training, as DISCUS parameters are not further updated and only VINN feature maps need to be kept in memory. Given the similar performance and factoring in the GPU requirements, we select the version with DISCUS pre-training followed by a segmentation training of VINN without further DISCUS updates for the state-of-the-art comparison. For those comparisons, we reallocate the freed-up GPU memory towards increased feature sizes in the hidden layers of DISCUS and VINN (see specification of the default method versus limited ablation setting in Section 3.2.2).

#### 4.1.2 Number of training participants

**Fig. 3.**
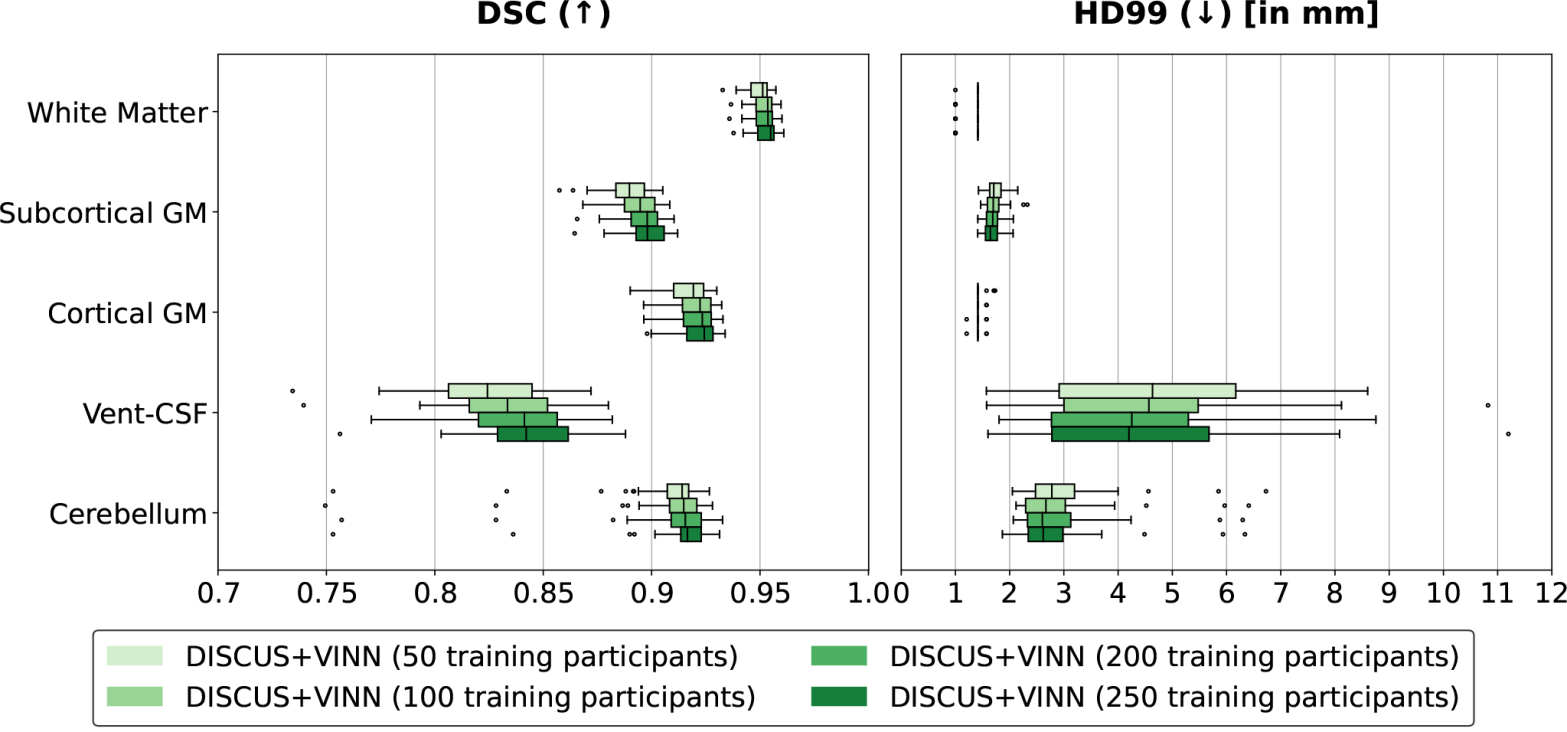
Ablation of numbers of training participants: Comparison of the segmentation performance for different numbers of training participants with our DISCUS+VINN method with pre-training evaluated on the HCP validation set. We compare the performance for two metrics: the Dice Similarity Coefficient (DSC) and the 99th percentile of the Hausdorff distance (HD99), across five region groups: White matter, subcortical and cortical gray matter (GM), ventricles and cerebrospinal fluid (Vent-CSF), and cerebellar regions, and with respect to FreeSurfer reference segmentations.

We also perform an ablation comparing different numbers of participants in the training set for the selected training strategy with DISCUS pre-training (see preceding subsection). For this comparison, we selected sex-balanced, nested training sets containing 50, 100, 200, and 250 participants. Each set also contains the 20 participants of the DISCUS pre-training. In addition, we have controlled for the number of training iterations/gradient updates and learning rate updates. In Figure 3, we observe a gradual performance increase with more participants being included in the training, but these increases seem to saturate, and we observe only small differences between networks trained with 200 and 250 training participants. Considering the performance saturation and system memory constraints, we choose to obtain the training sets from 250 participants for the state-of-the-art comparison.

### 4.2 State-of-the-art comparison

In this section, we compare our DISCUS+VINN method with state-of-the-art reference methods. DISCUS+VINN and DeepAnat are trained on the same HCP training set and we compare their performance for several q-space samplings on the HCP test set. In addition, we evaluate the methods on separate test sets not used for training: the in-house and ADNI datasets. These external datasets were not seen by methods during training or ablation/model selection. On the in-house test set, we compare our method with DeepAnat, DDParcel (as trained by the authors), and SynthSeg (as trained by the authors). Finally, on the ADNI dataset, we compare our method with DeepAnat and DDParcel (as trained by the authors). As SynthSeg was partially trained on ADNI data, we cannot rule out an overlap of the training set and our test set, and omit SynthSeg from the comparison.

#### 4.2.1 Human Connectome Project

**Fig. 4.**
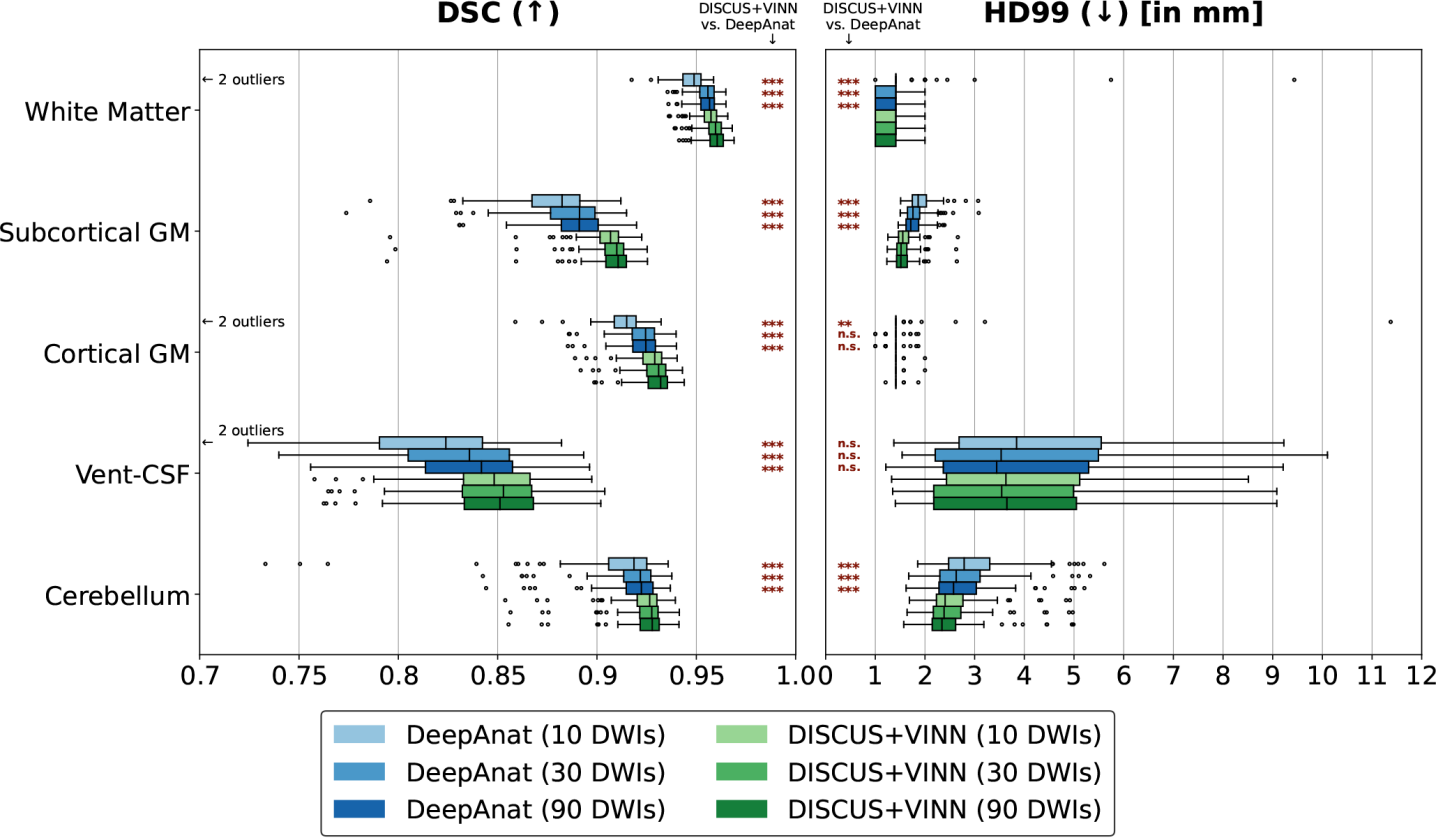
Method Comparison on the HCP dataset: Comparison of the segmentation performance across q-space samplings on the HCP test set for DeepAnat (shades of blue) and our DISCUS+VINN method (shades of green). Each method generates a segmentation for an input consisting of a mean $b_{0}$ image and a selection of 10 (light shade), 30 (medium shade), and 90 DWIs (dark shade) representing sparse, medium- and high-quality acquisitions. We compare the performance for two metrics: the Dice Similarity Coefficient (DSC) and the 99th percentile of the Hausdorff distance (HD99), across five region groups: White matter, subcortical and cortical gray matter (GM), ventricles and cerebrospinal fluid (Vent-CSF), and cerebellar regions, and with respect to FreeSurfer reference segmentations. Per selection of DWIs, we perform a one-sided statistical significance test, representing the degree to which DISCUS+VINN outperforms DeepAnat with *** if p<0.001 , ** if 0.001≤p<0.01 , * if 0.01≤p<0.05 , and “n.s.” for “not significant” if p≥0.05 .

For the comparison, we train the DeepAnat and our DISCUS+VINN method once in their default settings and obtain segmentations for three different q-space samplings containing 10, 30, and 90 DWIs in addition to a mean $b_{0}$ image. For our DISCUS+VINN method, the different samplings can be used directly as input to the DISCUS encoder, and we obtain the corresponding segmentations with a single pass through the network. For the DeepAnat workflow, we obtain the DeepAnat input representation via a diffusion tensor fit on the respective q-space sampling, use those to obtain a synthesized T1w image with the trained model, and obtain the segmentation with FreeSurfer. For the DeepAnat segmentation from the sparse sampling of 10 DWIs, we observe one failure case, which we exclude from the evaluation for all methods and choices of DWIs in the main comparison (Fig. 4 and Supplementary Table S1)—skewing results in favor of DeepAnat.

For both methods, we observe high segmentation performance for all region groups (see Fig. 4 and Supplementary Table S1), with medians above 0.88 DSC and around and below 2.8 mm HD99, except for ventricles and cerebrospinal fluid (Vent-CSF), where we observe medians above 0.82 DSC and below 3.9 mm HD99. Furthermore, for both methods, we observe generally increasing segmentation quality with denser samplings in q-space (higher DSC and lower HD99), showing that information from q-space complements the information of the mean $b_{0}$ images, yielding higher segmentation quality when added. For both methods, the increase from 10 to 30 DWIs generally results in a larger increase in segmentation performance than the increase from 30 to 90 DWIs, suggesting a decreasing marginal performance effect of denser samplings. While this effect can be observed for both methods, the performance of our DISCUS+VINN varies substantially less across q-space samplings compared to DeepAnat.

The performance differences are particularly pronounced for the subcortical gray matter and Vent-CSF regions (DSC) and for the sparse sampling of 10 DWIs. We also note that our DISCUS+VINN method, evaluated on the sparse input (10 DWIs), still outperforms DeepAnat evaluated on the dense input (90 DWIs), except for HD99 on Vent-CSF and ties for HD99 on Cortical GM and WM. For each q-space sampling, we perform statistical significance tests (see Section 3.4.1), showing that our DISCUS+VINN method significantly outperforms the state-of-the-art method DeepAnat on all region groups, samplings, and metrics with very few exceptions, i.e., Vent-CSF and Cortical GM for HD99. Our method combines a substantially smaller performance variance over q-space samplings with an overall high performance level, illustrating that DISCUS+VINN can consistently extract relevant information from the DWIs – including particularly very small numbers of DWIs.

In the Supplementary Material, we provide additional evaluations focusing on special aspects. In Supplementary Figure S2, we compare the methods’ segmentation performance when evaluated with reference labels obtained with FastSurfer (Henschel et al., 2020, 2022) instead of FreeSurfer. In Supplementary Figure S3, we compare the methods’ performance on two different samplings of 30 DWIs.

#### 4.2.2 External validation on the in-house dataset

**Fig. 5.**
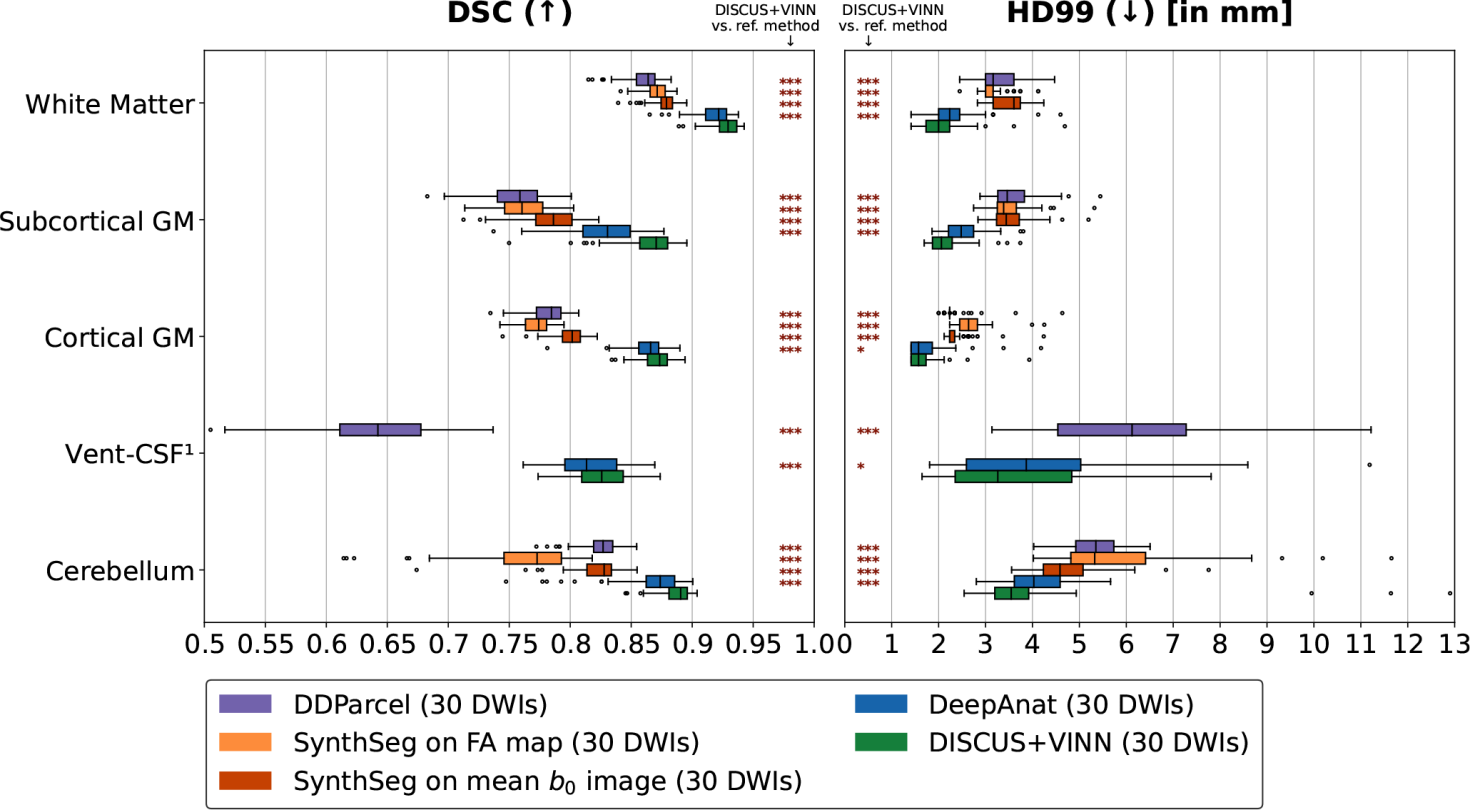
Method Comparison on the in-house dataset: Comparison of the segmentation performance on the in-house test set for DDParcel (purple), SynthSeg applied to FA maps (orange) and mean $b_{0}$ images (red), DeepAnat (blue), and our DISCUS+VINN method (green). Each method generates a segmentation for an input consisting of a mean $b_{0}$ image and 30 DWIs. Note that SynthSeg applied to mean $b_{0}$ images does not use the DWIs. We compare the performance for two metrics: the Dice Similarity Coefficient (DSC) and the 99th percentile of the Hausdorff distance (HD99), across five region groups: White matter, subcortical and cortical gray matter (GM), ventricles and cerebrospinal fluid (Vent-CSF), and cerebellar regions, and with respect to FreeSurfer reference segmentations. We perform one-sided statistical significance tests comparing our DISCUS+VINN method and each reference method, representing the degree to which DISCUS+VINN outperforms the reference method with *** if p<0.001 , ** if 0.001≤p<0.01 , * if 0.01≤p<0.05 , and “n.s.” for “not significant” if p≥0.05 . ^1^For SynthSeg, we do not report performance on Vent-CSF regions, as its label definitions cannot be aligned with FreeSurfer’s, making a direct comparison infeasible.

In this evaluation, we determine how the DISCUS+VINN, DeepAnat, DDParcel, and SynthSeg methods generalize to the in-house dataset—a separate test-set that was not used for training for any of the methods. We show quantitative results in the form of boxplots in Figure 5 and a results table in Supplementary Table S2 and provide a qualitative comparison in Supplementary Figure S1, showing segmentation slices for the different methods. For the DeepAnat method, we observe one failure case, which we exclude from the evaluation for all methods—skewing results in favor of DeepAnat. Compared to the HCP test set, the overall segmentation performance is slightly lower, but the results on the two datasets are not directly comparable as both datasets differ in spatial resolutions (1.5 mm vs. 1.25 mm for HCP), signal acquisition strategies (see single vs. dual phase encoding in Section 3.1.1.2), and we observed the presence of regions labeled as outliers by the Eddy tool during the preprocessing phase for the in-house dataset.

We observe very good performance for the DeepAnat and DISCUS+VINN methods. In contrast, DDParcel and both SynthSeg approaches yield less favorable segmentation results. In summary, our DISCUS+VINN method significantly outperforms all methods across regions and metrics.

#### 4.2.3 External validation on the ADNI dataset

**Fig. 6.**
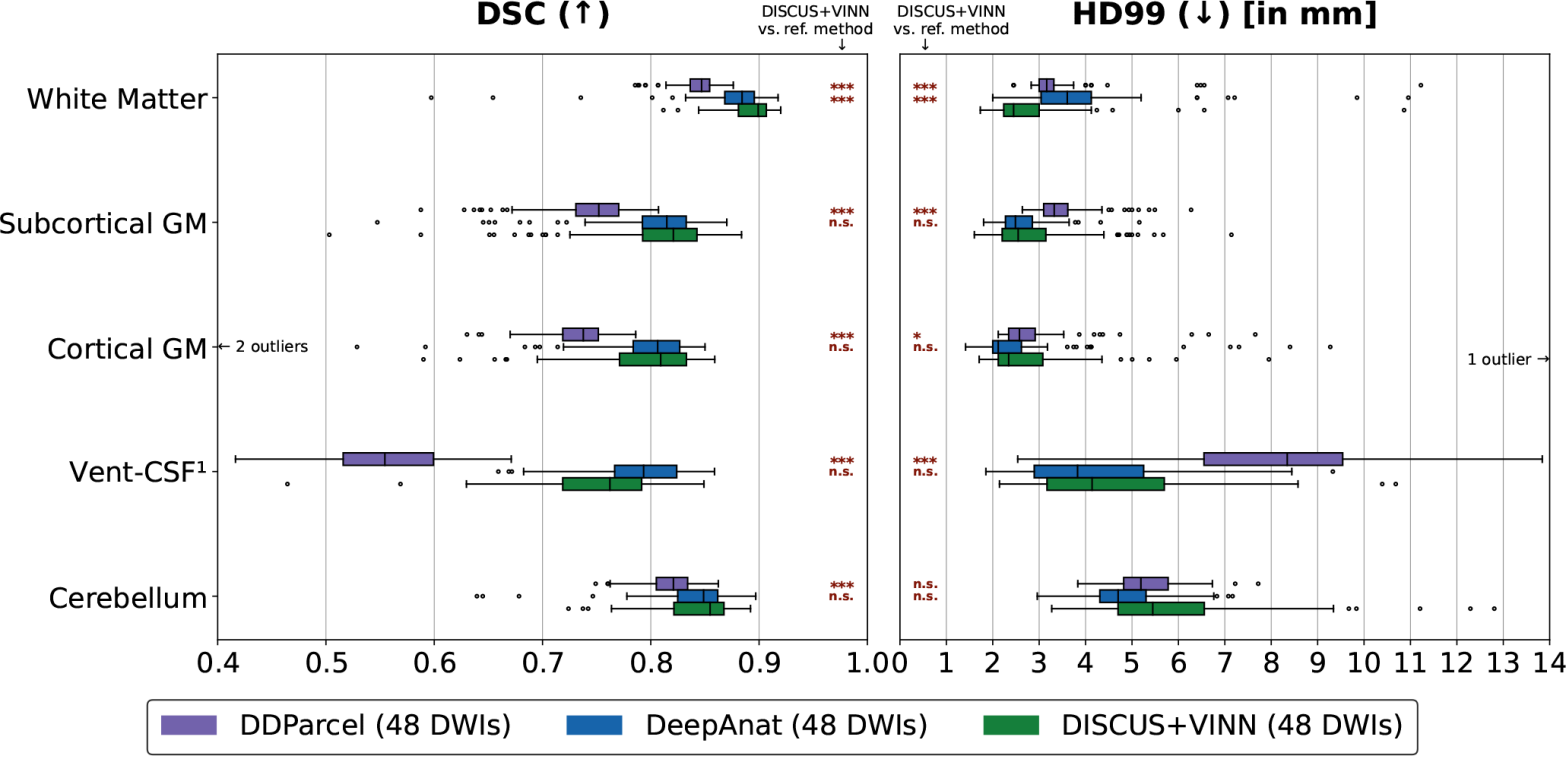
Method Comparison on the ADNI dataset containing different scanner manufacturers, condition groups, and acquisition parameters: Comparison of the segmentation performance on the ADNI for DDParcel (purple), DeepAnat (blue), and our DISCUS+VINN method (green). We compare the performance for two metrics: the Dice Similarity Coefficient (DSC) and the 99th percentile of the Hausdorff distance (HD99), across five region groups: White matter, subcortical and cortical gray matter (GM), ventricles and cerebrospinal fluid (Vent-CSF), and cerebellar regions, and with respect to FreeSurfer reference segmentations. We perform one-sided statistical significance tests comparing our DISCUS+VINN method and each reference method, representing the degree to which DISCUS+VINN outperforms the reference method with *** if p<0.001 , ** if 0.001≤p<0.01 , * if 0.01≤p<0.05 , and “n.s.” for “not significant” if p≥0.05 . ^1^As DDParcel failed to segment the inferior lateral ventricle in 8/126 cases, we obtained the Vent-CSF HD average from the other regions for the affected cases. This slightly biases the HD results for Vent-CSF in favor of DDParcel.

We perform an additional evaluation on the ADNI dataset, containing scans acquired with different scanners and acquisition parameters from participants in different condition groups (see Section 3.1.1.3 for details). On this dataset, accurate segmentation can be considered more challenging, considering lower spatial resolutions of diffusion data (2.0 mm vs. 1.25 mm for HCP), EPI artifacts in the raw data, and regions labeled as outliers by the Eddy tool in the preprocessing phase. In this evaluation, we compare our method with DeepAnat and DDParcel, that is, the methods for which the possibility of an overlap between our selected ADNI dataset and the training data of the respective method can be excluded. For the DeepAnat method, we observe one failure case, which we exclude from the evaluation for all methods. The DDParcel method fails to segment the left or right inferior lateral ventricle (or both) in 8/126 cases (part of Vent-CSF). These lower the average Dice Score for this method on the Vent-CSF region (see Fig. 6, left). For these cases, we obtain average Hausdorff distances for Vent-CSF based on the remaining segmented regions (in Vent-CSF), and use these averages in the boxplots and significance tests in Figure 6 (right) and Supplementary Table S3, slightly biasing the Hausdorff distance for Vent-CSF in favor of DDParcel.

We show the results in Figure 6 and in Supplementary Table S3. While our method significantly outperforms DDParcel across metrics and region groups with one exception (HD99, Cerebellum), we observe a similar performance for DISCUS+VINN and DeepAnat, except for the white matter, where DISCUS+VINN outperforms DeepAnat significantly. In addition to the pooled evaluation across all participants, we have performed stratified evaluations to assess factor-specific performance in Supplementary Section S4 by sex (see Supplementary Fig. S4), scanner manufacturer (see Supplementary Fig. S5), and condition group (see Supplementary Fig. S6).

## 5 Conclusion

This paper presents the DISCUS+VINN method that generates high-quality segmentations for heterogeneous raw dMRI acquisitions, which differ tremendously across datasets in the number of DWIs, order of measurements, q-space acquisition schemes, and details of the b-vectors and b-values. More specifically, the DISCUS component of our method learns to convert q-space information from a per-measurement basis in unstructured raw data containing a flexible number of images into a well-structured representation with a fixed number of channels and a consistent channel structure, which CNN-based segmentation networks rely on. The VINN component (a CNN) generates the segmentation based on a mean $b_{0}$ image and the DISCUS diffusion embedding. Combined, both components yield one network that can be applied directly to the unstructured raw data to generate a segmentation instead of relying on the intermediate fit of diffusion signal models such as the diffusion tensor.

We compare our method with the state-of-the-art methods for diffusion MRI segmentation, DeepAnat and DDParcel, which both rely on intermediate model fits with the diffusion tensor. In addition, we compare with SynthSeg, a method capable of segmenting any MRI contrast, applied to mean $b_{0}$ images and FA maps. On data from the Human Connectome Project, we have compared our DISCUS+VINN method with DeepAnat for sparse, medium- and high-quality samplings of q-space. In that comparison, we found strong statistical evidence that our DISCUS+VINN method outperforms DeepAnat for almost all region groups, q-space samplings, and performance metrics. Our method also shows less performance variance over q-space samplings compared to DeepAnat. When compared with reference segmentations generated with FastSurfer instead of FreeSurfer, the gap in segmentation performance widens for almost all region groups and metrics in favor of our DISCUS+VINN method. We also evaluate the performance of the methods on datasets not seen during training, which include scans acquired with multiple scanner models, spatial resolutions, and phase-encoding strategies, as well as data from populations far outside of the training distribution in terms of age and health status. On the in-house dataset, our method significantly outperforms all reference methods, that is, DeepAnat, DDParcel, and SynthSeg applied to mean $b_{0}$ images or FA maps. On the challenging ADNI dataset, our method significantly outperforms DDParcel on all but one region-metric pair and performs overall similarly to DeepAnat. For the white matter, our method significantly outperforms DeepAnat. Across the comparisons on these three datasets, DISCUS+VINN outperforms the second-best method, DeepAnat, with a high degree of statistical significance for many dataset-region-metric combinations. Furthermore, some results may potentially be biased in favor of DeepAnat, as both the reference and the DeepAnat segmentations are obtained with FreeSurfer. In addition, the DeepAnat method relies on the FreeSurfer software to generate segmentations, which i) takes a long time (several hours vs. a few minutes for our method) and ii) has proven error-prone, where FreeSurfer still fails to generate the segmentation based on the DeepAnat-generated synthetic T1w images after several modifications to reduce processing failures. In contrast, our DISCUS+VINN method (and pipeline) quickly segments with no observed processing failures.

Our DISCUS+VINN method can be easily extended to other q-space domains beyond a single shell, which we chose to provide a fair comparison with DeepAnat and DDParcel. Given suitable training data, DISCUS models can be trained for other sampling schemes, for example, multi-shell or grid-based patterns (see Ewert et al., 2024). Although the increased memory requirements associated with these acquisitions would generally pose a challenge, our two-phase training approach makes them feasible. While our evaluations indicate performance saturation for denser samplings at $b_{1000}$ , measurements at higher b-values offer increased tissue contrast—despite lower signal-to-noise ratios—which could enhance segmentation performance even further. Regardless of possible extensions, with a focus on the shell at $b_{1000}$ , which was acquired for many datasets, our model is widely applicable and generates high-quality segmentations leveraging diffusion measurements from diverse acquisitions, generally surpassing the quality of the state-of-the-art methods. Our DISCUS+VINN method can supply dMRI workflows, such as region-of-interest analyses or anatomically-constrained tractography, with very accurate segmentations from a variety of q-space sampling contributing to an increased accuracy in the analysis of the brain’s microstructure and connectivity.

## Supplementary Material

Supplementary Material

## Data Availability

In this paper, we use MRI data of two public datasets, the Young Adult dataset of the Human Connectome Project (HCP), available at https://balsa.wustl.edu, and the dataset of the Alzheimer’s Disease Neuroimaging Initiative (ADNI), available at https://ida.loni.usc.edu. In addition, we use MRI data of the Rhineland Study which is not publicly available due to data protection regulations. Access can be provided to scientists in accordance with the Rhineland Study’s Data Use and Access Policy. Requests to access the data should be directed to Monique M.B. Breteler at RS-DUAC@dzne.de. The source code for our method can be found on the website https://github.com/Deep-MI/DISCUSseg.

## Footnotes

1
https://github.com/Deep-MI/FastSurfer

2
https://github.com/liziyu0929/DeepAnat

3
https://github.com/zhangfanmark/DDParcel
