# Supplementary material for: Learning-based segmentation of diffusion-weighted MR images with arbitrary q-space samplings
Source: Imaging Neurosci (Camb). 2026 Jun 2;4:IMAG.a.1183. doi: 10.1162/IMAG.a.1183 (PMC13231284; doi:10.1162/IMAG.a.1183)
Supplement: Supplementary Material [file IMAG.a.1183_supp.pdf]

Supplementary Material for: Learning-based Segmentation of Diffusion-Weighted MR Images with Arbitrary *q*-Space Samplings

Christian Ewert,[1] David Kügler,[1] Martin Reuter[1,2,3,*]

[1]German Center for Neurodegenerative Diseases (DZNE), Bonn, Germany

[2]A. A. Martinos Center for Biomedical Imaging, Massachusetts General Hospital, Boston, MA, USA

[3]Department of Radiology, Harvard Medical School, Boston, MA, USA

[*]Correspondence: martin.reuter@dzne.de

March 3, 2026

S1 Result Tables

In addition to the boxplots shown in Section 4.2, we provide the results in table format showing the mean and standard deviation (SD) of metrics for each region group and method.

S1.1 HCP Dataset

Table S1: Segmentation performance on the HCP dataset: Dice Similarity Coefficient (mean ± SD, upper block) and Hausdorff distance (99th percentile, mm, mean ± SD, lower block)

Metric	Method	White Matter	Subcortical GM	Cortical GM	Vent-CSF	Cerebellum
Dice ↑	DeepAnat (10)	0.942 ± 0.038	0.877 ± 0.020	0.902 ± 0.085	0.816 ± 0.040	0.909 ± 0.033
	DeepAnat (30)	0.955 ± 0.006	0.886 ± 0.021	0.923 ± 0.010	0.830 ± 0.032	0.918 ± 0.017
	DeepAnat (90)	0.956 ± 0.006	0.889 ± 0.017	0.923 ± 0.010	0.836 ± 0.029	0.919 ± 0.016
	DISCUS+VINN (10)	**0.956 ± 0.006**	**0.904 ± 0.015**	**0.927 ± 0.009**	**0.846 ± 0.027**	**0.923 ± 0.013**
	DISCUS+VINN (30)	**0.959 ± 0.006**	**0.907 ± 0.015**	**0.929 ± 0.009**	**0.849 ± 0.028**	**0.924 ± 0.013**
	DISCUS+VINN (90)	**0.960 ± 0.006**	**0.908 ± 0.015**	**0.930 ± 0.009**	**0.849 ± 0.028**	**0.925 ± 0.013**
HD ↓	DeepAnat (10)	1.61 ± 0.94	1.92 ± 0.27	1.56 ± 1.02	4.25 ± 1.96	3.01 ± 0.77
	DeepAnat (30)	1.33 ± 0.22	1.81 ± 0.26	**1.41 ± 0.11**	3.97 ± 2.01	2.78 ± 0.70
	DeepAnat (90)	1.32 ± 0.23	1.77 ± 0.21	**1.40 ± 0.11**	3.87 ± 1.88	2.74 ± 0.68
	DISCUS+VINN (10)	**1.31 ± 0.20**	**1.59 ± 0.19**	**1.42 ± 0.06**	**3.94 ± 1.82**	**2.56 ± 0.58**
	DISCUS+VINN (30)	**1.22 ± 0.22**	**1.56 ± 0.19**	1.42 ± 0.06	**3.76 ± 1.75**	**2.50 ± 0.61**
	DISCUS+VINN (90)	**1.19 ± 0.22**	**1.55 ± 0.19**	1.42 ± 0.05	**3.80 ± 1.82**	**2.48 ± 0.62**

S1.2 In-house Dataset

Table S2: Segmentation performance on the in-house dataset: Dice Similarity Coefficient (mean ± SD, upper block) and Hausdorff distance (99th percentile, mm, mean ± SD, lower block) [1] Substantially different label definitions make a comparison with SynthSeg on Vent-CSF infeasible.

Metric	Method	White Matter	Subcortical GM	Cortical GM	Vent-CSF[1]	Cerebellum
	DDParcel	0.861 ± 0.012	0.755 ± 0.024	0.782 ± 0.015	0.641 ± 0.049	0.825 ± 0.015
	SynthSeg on FA	0.871 ± 0.009	0.760 ± 0.022	0.772 ± 0.013		0.762 ± 0.044
Dice ↑	SynthSeg on b_0	0.878 ± 0.009	0.784 ± 0.022	0.800 ± 0.012		0.822 ± 0.023
	DeepAnat	0.919 ± 0.014	0.829 ± 0.028	0.863 ± 0.015	0.815 ± 0.027	0.868 ± 0.027
	DISCUS+VINN	**0.928 ± 0.011**	**0.864 ± 0.023**	**0.870 ± 0.012**	**0.826 ± 0.022**	**0.887 ± 0.012**
	DDParcel	3.28 ± 0.39	3.57 ± 0.44	2.28 ± 0.31	6.21 ± 1.90	5.31 ± 0.60
	SynthSeg on FA	3.14 ± 0.27	3.48 ± 0.40	2.66 ± 0.33		5.77 ± 1.39
HD ↓	SynthSeg on b_0	3.48 ± 0.32	3.52 ± 0.39	2.34 ± 0.27		4.72 ± 0.70
	DeepAnat	2.33 ± 0.45	2.55 ± 0.42	1.72 ± 0.41	4.04 ± 1.76	4.13 ± 0.65
	DISCUS+VINN	**1.96 ± 0.47**	**2.15 ± 0.39**	**1.67 ± 0.32**	**3.72 ± 1.58**	**3.80 ± 1.50**

S1.3 ADNI Dataset

Table S3: Segmentation performance on the ADNI dataset: Dice Similarity Coefficient (mean ± SD, upper block) and Hausdorff distance (99th percentile, mm, mean ± SD, lower block)
[1] As DDParcel failed to segment the inferior lateral ventricle in 8/126 cases, we obtained the Vent-CSF HD average from the other regions for the affected cases. This slightly biases the HD results for Vent-CSF in favor of DDParcel.

Metric	Method	White Matter	Subcortical GM	Cortical GM	Vent-CSF[1]	Cerebellum
	DDParcel	0.843 ± 0.017	0.746 ± 0.039	0.733 ± 0.028	0.557 ± 0.057	0.817 ± 0.024
Dice ↑	DeepAnat	0.876 ± 0.041	0.804 ± 0.048	0.788 ± 0.092	**0.790 ± 0.043**	0.839 ± 0.041
	DISCUS+VINN	**0.894 ± 0.019**	**0.807 ± 0.058**	**0.795 ± 0.051**	0.751 ± 0.058	**0.842 ± 0.036**
	DDParcel	3.35 ± 0.93	3.46 ± 0.60	2.77 ± 0.81	8.21 ± 2.26	5.28 ± 0.70
HD ↓	DeepAnat	3.73 ± 1.26	**2.62 ± 0.50**	**2.51 ± 1.19**	**4.16 ± 1.61**	**4.85 ± 0.82**
	DISCUS+VINN	**2.84 ± 0.99**	2.87 ± 0.94	2.84 ± 2.03	4.58 ± 1.81	5.87 ± 1.66

S2 Qualitative Comparison

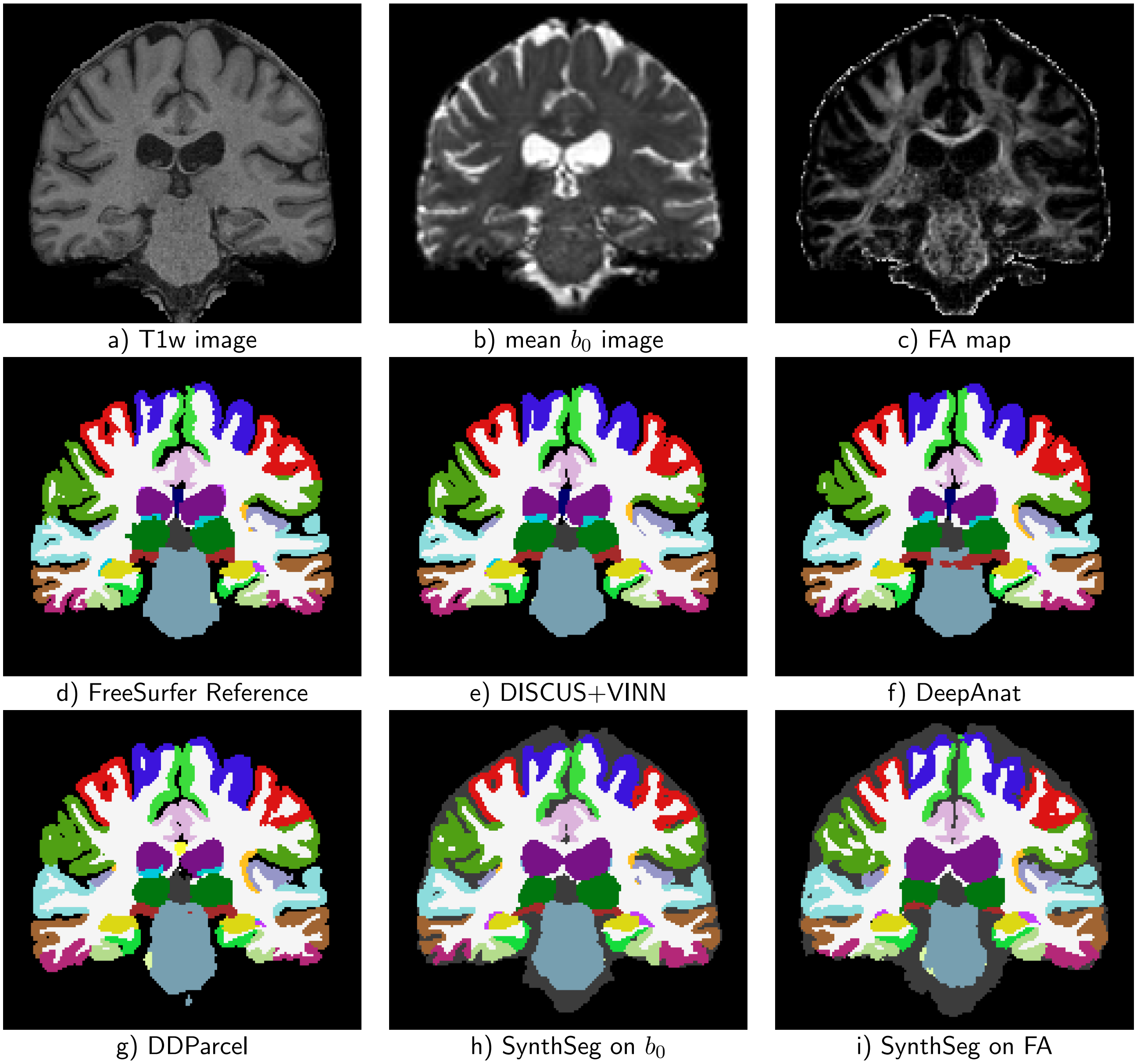

Figure S1: Qualitative method comparison on coronal brain slices of a participant of the in-house dataset. In the top left, we show the T1w image (a), which is used by FreeSurfer to generate the reference segmentation (d). Also in the top row, we show the mean b_0 image (b) and a fractional anisotropy (FA) map (c), as examples of the dMRI acquisition. Following the reference segmentation in d), we show the segmentations of all methods in their native label spaces (e-i). For a fair comparison, labels are brought into correspondence, with one exception (SynthSeg's CSF definition, see gray label surrounding the brain in h) and i)).

S3 Additional Evaluations on the HCP Dataset

S3.1 FreeSurfer vs. FastSurfer Reference

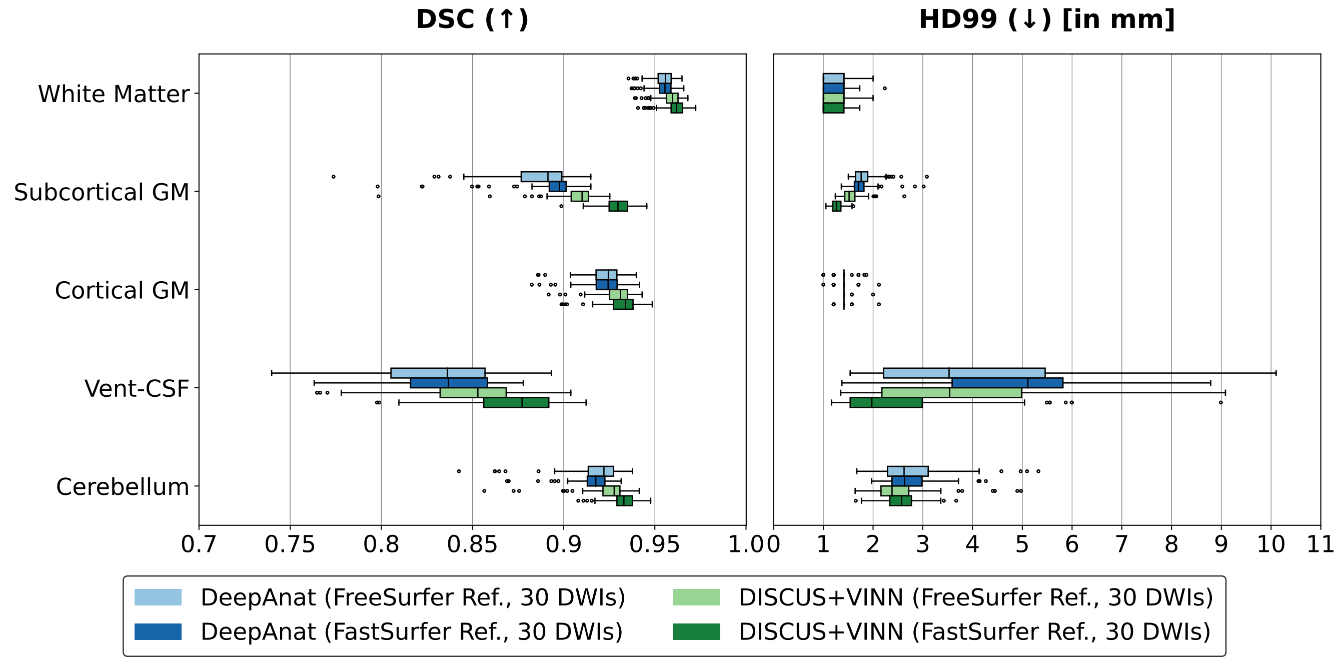

Figure S2: Types of Reference Segmentations: Comparison of segmentation performance of DeepAnat (blue shades) and our DISCUS+VINN method (green shades) when evaluated with FreeSurfer-based (light shade) and FastSurfer-based reference segmentations (dark shade). Each method generates a segmentation for an input consisting of a mean b_0 image and 30 DWIs. We compare the performance for two metrics: the Dice Similarity Coefficient (DSC) and the 99th percentile of the Hausdorff distance (HD99), and across five region groups: White matter, subcortical and cortical gray matter (GM), ventricles and cerebrospinal fluid (Vent-CSF), and cerebellar regions.

In this comparison, we evaluate the performance with respect to FastSurfer reference segmentations as opposed to segmentations from FreeSurfer (see Figure S2). Compared with FreeSurfer, FastSurfer has demonstrated improved test-retest reliability and more statistically robust results for cortical thickness analyses (Henschel et al. 2020, Henschel and Kügler et al., 2022). When changing the reference from FreeSurfer to FastSurfer, DeepAnat's performance remains overall similar, with three exceptions (subcortical GM and cerebellar areas for DSC and Vent-CSF regions for HD99). In contrast, our DISCUS+VINN method shows consistent improvements across all region groups (with few exceptions for HD99) – implying that DISCUS+VINN independently learned similar patterns to FastSurfer. Overall, the performance gap between DeepAnat and DISCUS+VINN widens.

S3.2 Sampling 30 DWIs vs. 30* DWIs on the HCP dataset

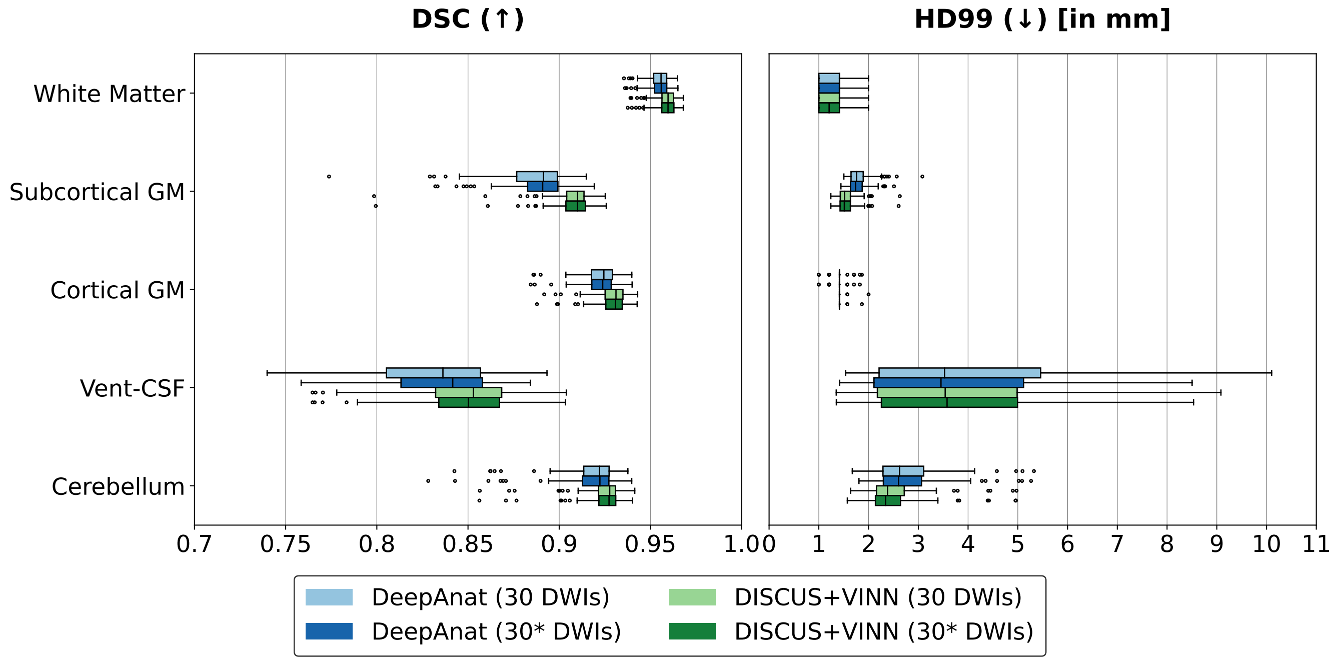

Figure S3: Sampling schemes 30 DWIs vs 30* DWIs: Comparison of segmentation performance of DeepAnat (blue shades) and our DISCUS+VINN method (green shades) evaluated for segmentations based on inputs for two different samplings of 30 DWIs. The sampling *30 DWIs* (light shade) was obtained by us, aiming to maximize the angular coverage of the shell, whereas the sampling *30* DWIs* (dark shade) is proposed in the DeepAnat publication and is used to train DeepAnat. We compare the performance for two metrics: the Dice Similarity Coefficient (DSC) and the 99th percentile of the Hausdorff distance (HD99), across five region groups: White matter, subcortical and cortical gray matter (GM), ventricles and cerebrospinal fluid (Vent-CSF), and cerebellar regions, and with respect to FreeSurfer reference segmentations.

In this evaluation, we analyze how different q-space samplings containing the same number of samples impact method performance. In particular, the sampling referred to as *30 DWIs* maximizes the angular coverage of b-vectors on the sphere, whereas the sampling *30* DWIs* was used to train DeepAnat (for details, see Section 3.4.2). The results shown in Figure S3 suggest a slight improvement of DeepAnat on the 30* sampling for Vent-CSF regions, but overall, both method's performance for the two samplings is very similar.

S4 Stratified Evaluations for the ADNI dataset

In addition to the evaluation pooling all participants of our ADNI dataset (see Section 4.3.2 and Figure 6), we performed stratified evaluations showing performance by sex, scanner manufacturers, and condition group, to determine patterns in the similarity of method-generated segmentations and their reference counterparts across methods, metrics, and regions. Looking at evaluations per sex (female, male), we observe a very slight bias towards higher similarity for female as opposed to male participants (see Figure S4). For scanner manufacturers, we do not observe a clear pattern (see Figure S5), and for the condition groups (see Figure S6), the similarity can be ranked by condition group from largest to smallest: 1) healthy controls, 2) mild cognitive impairment, and 3) Alzheimer's disease. These differences are likely caused by i) data quality/registration and ii) domain shift. On one hand, cortical atrophy, ventricular enlargement, and white-matter damage may make an accurate registration more challenging, leading to local distortions in alignment between T1w-based reference segmentations and diffusion MRI-based segmentations, yielding less favorable metrics. In addition, participant motion in the scanner has been found to differ systematically between AD/MCI and healthy controls (Haller et al., 2014), which may have contributed to the pattern via slightly poorer data quality, also contributing to less accurate registrations. On the other hand, the methods themselves may also contribute to this pattern, as all three methods were trained exclusively on the HCP Young Adult dataset, containing only young and healthy participants, and being presented with substantially older individuals with MCI or AD constitutes a domain shift, possibly negatively impacting performance.

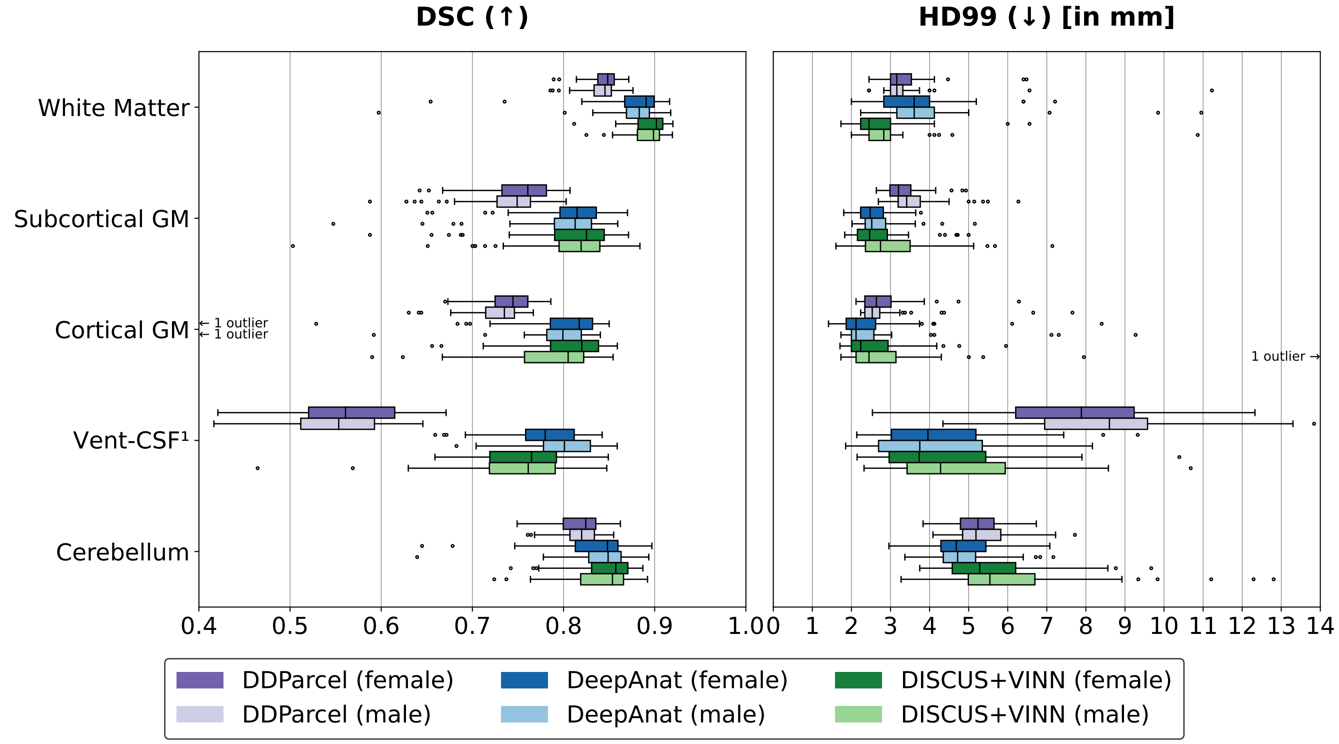

Figure S4: ADNI stratification by sex: Comparison of segmentation performance of DDParcel (purple shades), DeepAnat (blue shades), and our DISCUS+VINN method (green shades) evaluated per sex, i.e., female and male). We compare the performance for two metrics: the Dice Similarity Coefficient (DSC) and the 99th percentile of the Hausdorff distance (HD99), across five region groups: White matter, subcortical and cortical gray matter (GM), ventricles and cerebrospinal fluid (Vent-CSF), and cerebellar regions, and with respect to FreeSurfer reference segmentations.

[1] As DDParcel failed to segment the inferior lateral ventricle in 8/126 cases, we obtained the Vent-CSF HD average from the other regions for the affected cases. This slightly biases the HD results for Vent-CSF in favor of DDParcel.

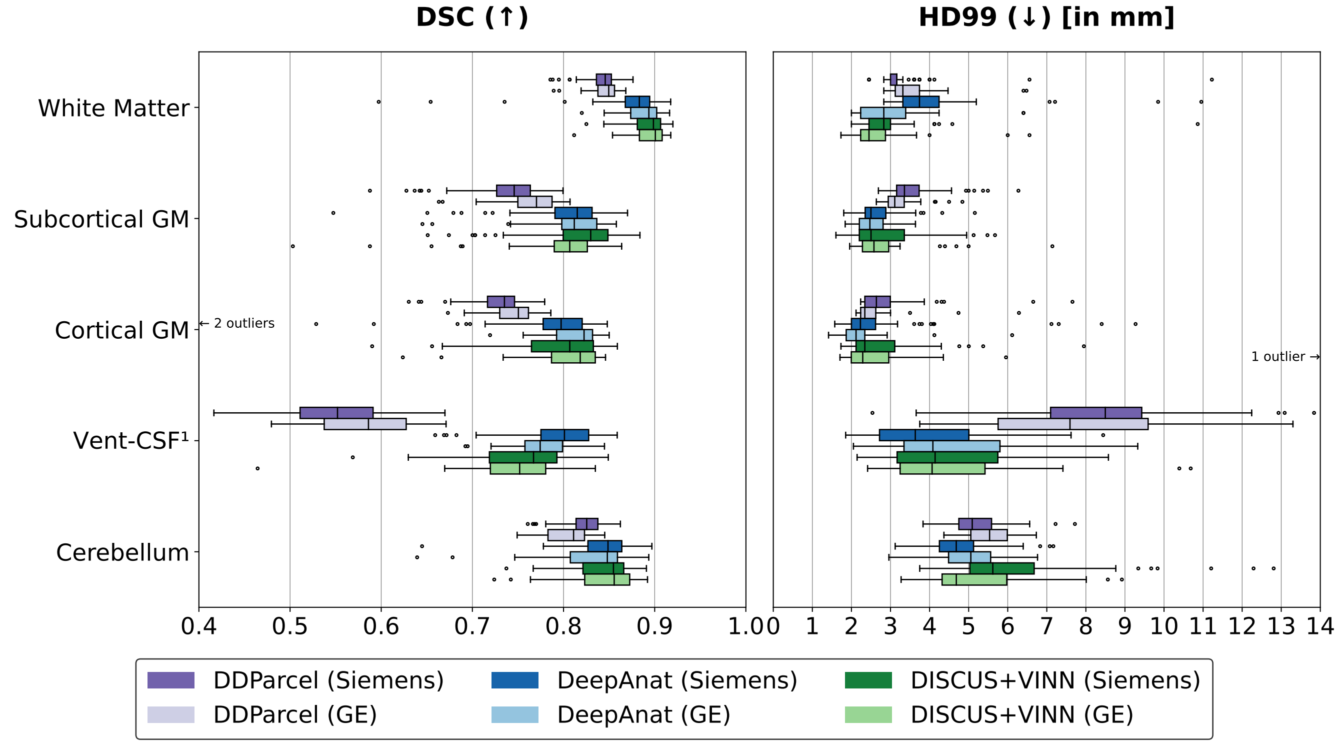

Figure S5: ADNI stratification by scanner manufacturer: Comparison of segmentation performance of DDParcel (purple shades), DeepAnat (blue shades), and our DISCUS+VINN method (green shades) evaluated per scanner manufacturer, i.e., Siemens and General Electric (GE). We compare the performance for two metrics: the Dice Similarity Coefficient (DSC) and the 99th percentile of the Hausdorff distance (HD99), across five region groups: White matter, subcortical and cortical gray matter (GM), ventricles and cerebrospinal fluid (Vent-CSF), and cerebellar regions, and with respect to FreeSurfer reference segmentations.

[1] As DDParcel failed to segment the inferior lateral ventricle in 8/126 cases, we obtained the Vent-CSF HD average from the other regions for the affected cases. This slightly biases the HD results for Vent-CSF in favor of DDParcel.

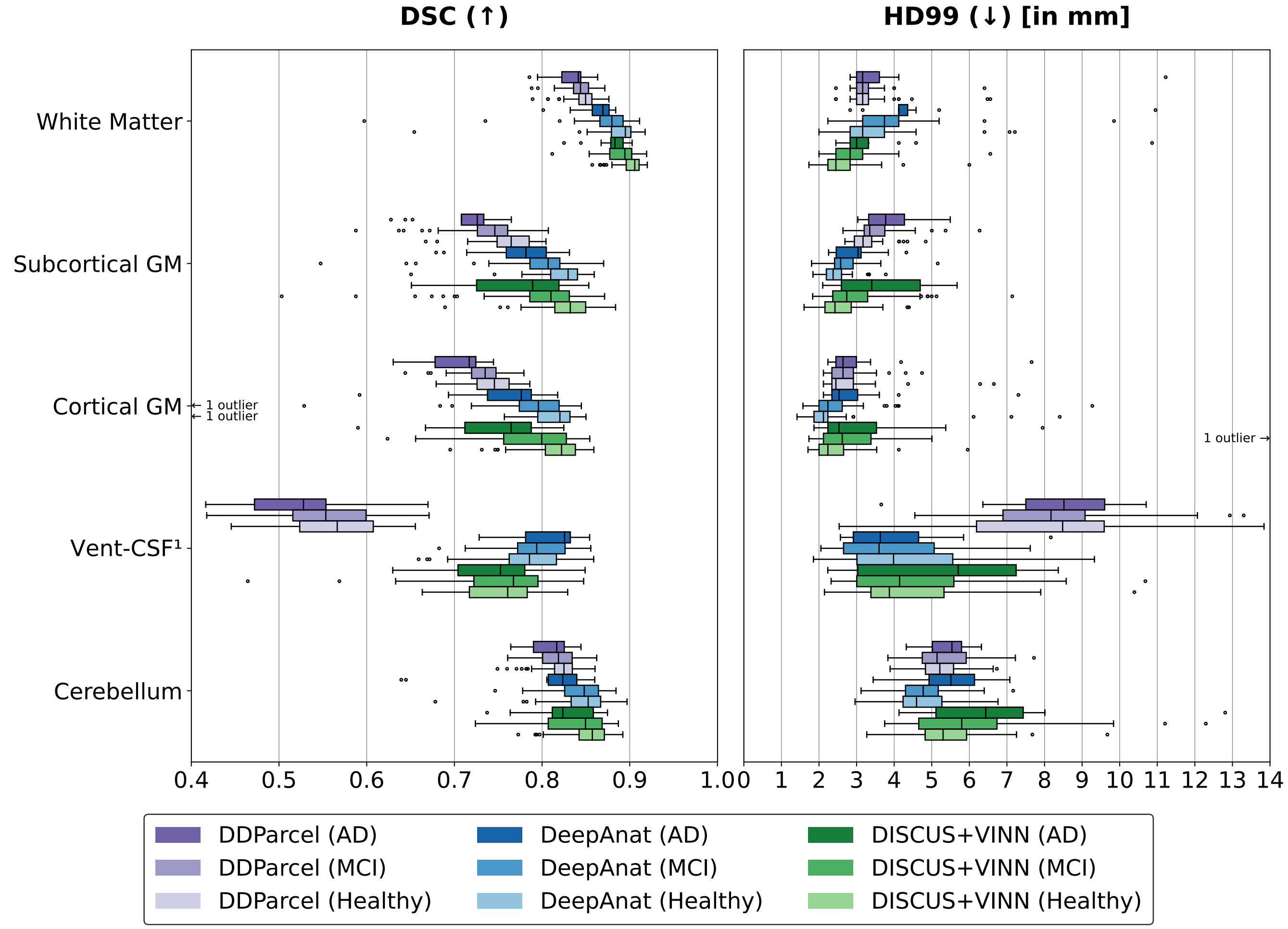

Figure S6: ADNI stratification by condition groups: Comparison of segmentation performance of DDParcel (purple shades), DeepAnat (blue shades), and our DISCUS+VINN method (green shades) evaluated per condition group, i.e., groups of participants with Alzheimer's disease (AD) or mild cognitive impairment (MCI), and healthy controls. We compare the performance for two metrics: the Dice Similarity Coefficient (DSC) and the 99th percentile of the Hausdorff distance (HD99), across five region groups: White matter, subcortical and cortical gray matter (GM), ventricles and cerebrospinal fluid (Vent-CSF), and cerebellar regions, and with respect to FreeSurfer reference segmentations.
[1] As DDParcel failed to segment the inferior lateral ventricle in 8/126 cases, we obtained the Vent-CSF HD average from the other regions for the affected cases. This slightly biases the HD results for Vent-CSF in favor of DDParcel.

References

Haller, S., Monsch, A. U., Richiardi, J., Barkhof, F., Kressig, R. W., & Radue, E.-W. (2014). Head motion parameters in fmri differ between patients with mild cognitive impairment and alzheimer disease versus elderly control subjects. *Brain Topography*, *27*, 801–807. https://doi.org/10.1007/s10548-014-0358-6

Henschel, L., Conjeti, S., Estrada, S., Diers, K., Fischl, B., & Reuter, M. (2020). Fastsurfer - a fast and accurate deep learning based neuroimaging pipeline. *NeuroImage*, *219*, 117012. https://doi.org/10.1016/j.neuroimage.2020.117012

Henschel, L., Kügler, D., & Reuter, M. (2022). FastSurferVINN: Building resolution-independence into deep learning segmentation methods—A solution for HighRes brain MRI. *NeuroImage*, *251*, 118933. https://doi.org/10.1016/j.neuroimage.2022.118933
